# Use of health promotion manga to encourage physical activity and healthy eating in Japanese patients with metabolic syndrome: a case study

**DOI:** 10.1186/s13690-018-0273-5

**Published:** 2018-06-18

**Authors:** Takashi Shimazaki, Munehiro Matsushita, Misa Iio, Koji Takenaka

**Affiliations:** 1 Department of Health & Physical Education, Faculty of Humanities, Sophia University, 7-1 Kioi-cho, Chiyoda-ku, Tokyo, Japan; 2 Department of Physical Education, Tokai University, 4-1-1 Kitakaname, Hiratsuka-shi, Kanagawa Japan; 3 College of Nursing, Kanto-Gakuin University, 1-50-1 Mutsuurahigashi, Kanazawa-ku, Yokohama, Japan; 4 Faculty of Human Sciences, Waseda University, 2-579-15 Mikajima, Tokorozawa, Saitama, Japan

**Keywords:** Physical activity, Eating behavior, Manga, Metabolic syndrome

## Abstract

**Background:**

The present case study investigated the feasibility of using manga (Japanese-style comic books) to promote physical activity and healthy eating behavior in Japanese patients with metabolic syndrome.

**Methods:**

A one-arm pre-post intervention was conducted in a Japanese suburban community. Twenty participants with a diagnosis of metabolic syndrome were recruited via health checkups. Health promotion manga were developed by the researcher, a publishing specialist, and a professional illustrator. We measured participants’ self-reported physical activity, eating behavior, and psychological readiness to change toward engaging in healthy behavior.

**Results:**

At 1 month after the intervention there were no significant differences in physical activity scores, but small positive changes in vigorous (*R*^*2*^ = 0.02) and moderate (*R*^*2*^ = 0.01) physical activity scores were observed. Total healthy eating behavior scores were significantly improved (*p* < 0.05, *R*^*2*^ = 0.47). In addition, participants reported positive change in psychological readiness, such as increased intention to engage in healthy behavior, enhanced self-efficacy, and benefits of using manga.

**Conclusions:**

This study demonstrates that manga interventions have potential to encourage healthy eating in patients with metabolic syndrome.

**Electronic supplementary material:**

The online version of this article (10.1186/s13690-018-0273-5) contains supplementary material, which is available to authorized users.

## Background

Metabolic syndrome and non-communicable diseases are serious health concerns globally [1] and in Japan [2]. Developing a healthy lifestyle, especially physical activity and healthy eating behavior, contribute to prevention of disease and health promotion [3, 4]. However, increasing knowledge, improving attitudes, and promoting behavior change in high-risk populations that lack self-efficacy and health literacy is difficult because of the low priority given to behavior change by those who lack self efficacy and health literacy [5].

Entertainment education is potentially an effective way to provide health information to high-risk populations [6, 7]. Entertainment education is defined as a purposeful communication strategy that provides educational information intended to increase knowledge and awareness via entertainment media, such as radio, television, popular music, films, digital games, and comics [8]. The efficacy of entertainment education has been confirmed, particularly in populations with low health literacy [9]. Several studies demonstrated that manga (Japanese-style comic books) had a positive influence on behavior change interventions in various health promotion setting (e.g., prevention of human immunodeficiency virus, schistosomiasis, lymphatic filariasis, cancer, smoking; and in nutrition education) [10, 11]. Manga interventions are supported by three psychological and behavioral theories: social cognitive theory of modeling characters engaging in health behavior [12]; a narrative or story telling approach to increase acceptability [13, 14]; and a graphic effect to enable sensory understanding [15].

Manga is a significant modern Japanese sub-culture [16, 17]. From a psycho-behavioral theoretical and cultural background, health promotion manga is also potentially acceptable media for high-risk populations with low health literacy in Japan [10]. However, the effectiveness of manga interventions has not been reported in a health promotion intervention in Japan. This case study aimed to confirm the positive influence of health promotion manga focused on increasing physical activity and healthy eating for patients diagnosed with metabolic syndrome.

## Methods

### Study design and participant recruitment

This case study was designed as a one-arm pre-post trial conducted in the local public health center in Tokigawa town, a suburban community in the midland of Saitama Prefecture, Japan, located 60 km northwest of Tokyo [18]. Participants were recruited during a specific health checkup with specific health guidance [19]. This is a mandatory nation-wide health checkup and health guidance to support early detection of risks for metabolic syndrome in people aged 40–75 years in Japan. The checkup aims to identify high-risk populations, and provides motivational counseling to change participants’ unhealthy lifestyles delivered by public health nurses [20]. The target population attended the specific health checkup for measurement of anthropometric indices (i.e., body mass index and abdominal circumference), blood pressure, blood exam (i.e., triglyceride, high density lipoprotein [HDL] and low density lipoprotein cholesterol, and hemoglobin A1c), hepatic function, and urinary examination [21]. After the initial screening, 150 patients were identified and recruited. Local public health nurses invited people at high-risk of metabolic syndrome to participate in this study via face-to-face recruitment. In total, 20 participants (response rate 13%) with a diagnosis of metabolic syndrome met the study inclusion criteria: (1) abdominal circumference of over 85 cm (males) or over 90 cm (females); (2) neutral lipid over 150 mg/dl or HDL cholesterol under 40 mg/dl; (3) blood pressure over 130/85 mmHg; and (4) fasting blood glucose over 110 mg/dl [22]. The exclusion criterion was participants who did not complete the post questionnaire. Twelve participants completed the pre-post questionnaire.

### Development of health promotion manga

Manga development was based on the theory of entertainment education, including the social cognitive theory of modeling [12], narrative approaches [13, 14], and graphic effects [15]. In addition, the content was developed using the small change model [23, 24]. Development of the health promotion manga, including illustrations and editing layout, was managed by a publishing company in Japan. The publishing director acted as an intermediary between the first author and the professional illustrator. The first author decided the storyline and images of the main characters.

**Fig. 1:**
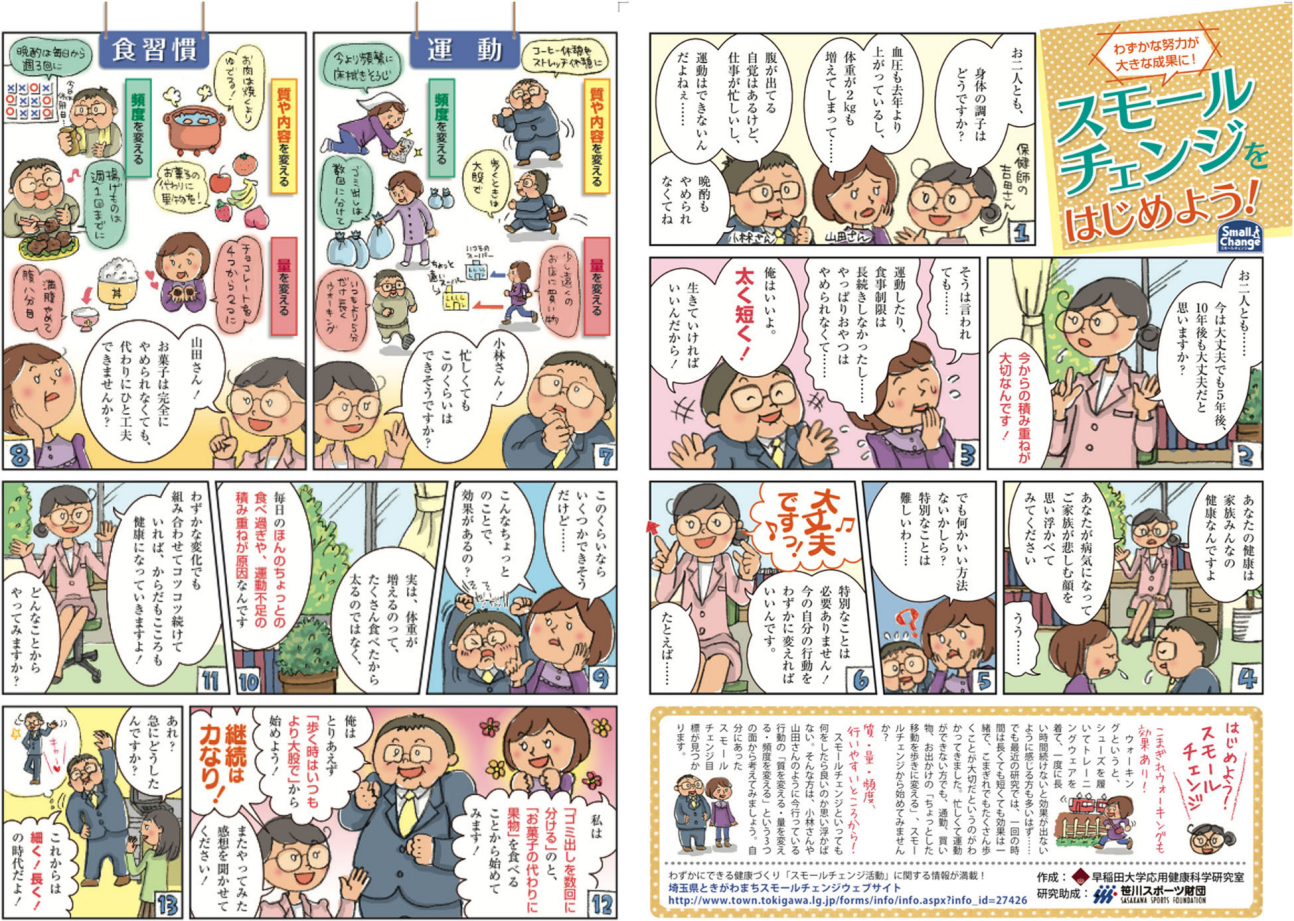
Health promotion manga to encourage healthy lifestyle. Showed health promotion manga that present study use to encourage physical activity and healthy eating

Figure 1 and Table 1 show the manga and dialogues. The characters were a public health nurse with two clients (male and female). The male client (Mr. Kobayashi) had no health concerns and no intention to engage in a healthy lifestyle, although he has an unhealthy lifestyle. In contrast, the female client (Mrs. Yamada) noticed that she needed to change her lifestyle, but does not understand how to change her lifestyle. The public health nurse, Yoshida, recommends small changes in their present lifestyles (detail of manga was shown in Additional file 1). After drafting a sample illustration for each character, the publishing director and illustrator discussed and modified the character’s concept, body shape, and terms. The total cost of producing the manga (including 500 prints) was almost 2700 USD (300,000 yen).

**Table 1:** Health promotion manga dialogue

Frame	Dialogue
1	*PHN Yoshida*: How is your health, both of you?
	*Mrs. Yamada:* Blood pressure has also increased since last year and weight has also increased by 2 kg…
	*Mr. Kobayashi*: I am aware that I have a paunch but I am busy with work and cannot exercise…and I have also not been able to stop drinking at dinner.
2	*PHN Yoshida*: Both of you…at present all is fine but do you think five years, ten years from now too all will be fine? It is important to make efforts from now onwards!
3	*Mrs. Yamada*: Even then …I have not been able to continue exercising and restricting my diet for a long time…also, I cannot really give up sweets…
	*Mr. Kobayashi*: I am fine. It’s all right to live short if I can do what I want to do!
4	*PHN Yoshida*: Your health is the health of all the members of your family. Please think of all the sad faces of your family members if you fall ill.
	*Mr. Kobayashi & Mrs. Yamada*: Hmm…
5	*Mrs. Yamada*: But isn’t there a good way of doing it? It is difficult to do something out of the way…
6	*PHN Yoshida*: Don’t worry! There is no need to use anything special! All you need to do is just change your everyday actions a little. For example…
7	*PHN Yoshida*: Mr. Kobayashi! Even though you are busy, will you be able to manage this much?
	[*Physical activity small change*] *Change the nature or content*: walk in long strides; coffee break into a stretch break; *Changing the quantity*: go shopping to a store that is a little further away; walk for 5 min longer than usual; *Change the frequency*: mop the floor clean more frequently than at present; divide the trash and put it out more often.
8	*PHN Yoshida*: Ms. Yamada! There is no need to stop eating sweets altogether, but do you think you can come up with a way of reducing intake of sweets?
	[*Healthy eating small change*] *Change the nature or content*: meat is better boiled rather than grilled; fruits instead of sweets; *Changing the quantity*: reduce chocolate to two pieces from four pieces; eat moderately and never fill your stomach; *Change the frequency*: reduce drinking at dinner from every day to three times a week; eat deep-fried food only once a week.
9	*Mrs. Yamada*: I think I may be able to manage this much…Will these small things be effective?
10	*PHN Yoshida*: Actually, weight does not increase because one has eaten too much. The cause is the piling up of the little bit of overeating and lack of exercise every day.
11	*PHN Yoshida*: Even small changes when continued consistently make both the body and mind healthy! What would you like to begin with?
12	*Mrs. Yamada*: I would like to divide the garbage and increase the number of times I go to put out the trash and start eating fruits instead of sweets!
	*Mr. Kobayashi*: For the time being, I would like to start with “walking in longer strides that usual”!
	*PHN Yoshida*: Perseverance will accomplish all things! Do let us know about your experiences!
13	*Co-worker*: What’s this? What’s happening all of a sudden?
	*Mr. Kobayashi*: The future is all about having a long, frugal life!

This study used a behavior change strategy based on the theory that small changes lead to comprehensive behavior change [23, 24]. Recommended health behaviors were selected from previous qualitative research that identified lifestyle behaviors in the healthy Japanese population [25, 26].

As shown in Table 2, descriptive tips for changing participants’ lifestyles were introduced. Topic one included the effect of brisk walking, based on results of a cross sectional study [27]. That study showed physical activity of less than 10-min duration was associated with cardiovascular health outcomes. The second topic was how to set a specific action plan based on previous small change practice literature [28].

**Table 2:** Tips to encourage behavior change

Topics	Description
Walking bit by bit is also effective!	Many people should feel that putting on shoes and training wear and going for a walk is not effective unless you take a long walk… However, recent research has shown that walking a lot, even a bit at a time, is important. This is due to the cumulative effect of both short and long walks. If you are someone who is busy and cannot exercise, how about starting with small changes? Such as, “changing brief trips into walks.”
Start from the easy things, such as quality, quantity and frequency!	It is difficult to come up with small changes. People who struggle to come up with something, like Kobayashi and Ms. Yamada, should start by considering their current behavior from three perspectives: “changing quality,” “changing quantity” and “changing frequency.” That way, you can identify a goal of small changes that suit you.

### Procedure

**Fig. 2:**
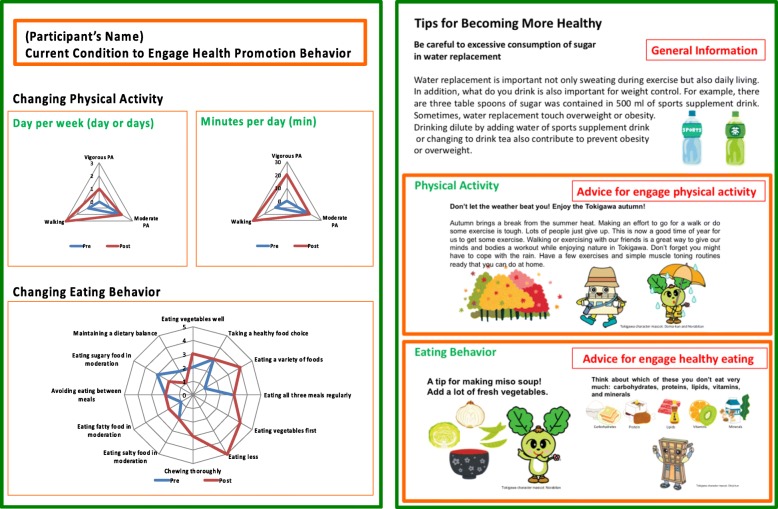
Personalized follow up letter. Showed English version of the personalized follow up letter

First, participants completed a questionnaire covering demographic characteristics and self-reported current physical activity and eating behavior. Next, a public health nurse distributed the health promotion manga, and provided instruction for planning at least one small lifestyle change action plan. After 1 month, a questionnaire along with a return envelope was mailed to each participant. After completion of the second questionnaire, the author mailed a personalized letter to each participant (Fig. 2, and Additional file 2) that included a 1-month change of health behavior plan and advice (Additional file 3) to support further improvement of participants’ lifestyles.

### Outcome measurement

Primary outcomes were changes in physical activity and healthy eating behavior. Physical activity was measured with the short form of the International Physical Activity Questionnaire (IPAQ) [29]. The IPAQ short form has acceptable reliability and validity (Spearman-Brown split half reliability and criterion-related validity via pedometer steps) [30]. Healthy eating behaviors were measured with the Healthy Eating Behavior Index for Japanese (HEBI-J) [26]. The HEBI-J was based on qualitative research among Japanese people that engage in healthy eating. The HEBI-J uses a five-point Likert-type scale ranging from “never” to “very often.” In addition, an index of “Balance,” “Pattern,” “Restriction,” and total scores was formed by combing four items for each factor and all 12 items. The internal validity of the relationship between the HEBI-J and behavior change stage for healthy eating was confirmed (balance: *r* = .64, *p* ≤ .01; pattern: *r* = .48, *p* ≤ .01; restriction: *r* = .35, p ≤ .01) [31]. The factor validity of the scale was tested using confirmatory factor analysis (GFI, .94; AGFI, .90; CFI, .90; RMSEA, .08; SRMR, .53). In addition, reliability was evaluated using Cronbach’s alpha coefficients (balance, .84; pattern, .63; restriction, .79) and split-half reliability analysis (*r* = .80, *p* ≤ .01).

Secondary outcomes were perceived health benefits from participating in the manga trial. After the 1-month trial, participants were administered an additional free-description questionnaire. Participants were asked, “Please tell us your impression of participating in the manga health promotion trial.” Participants were also asked if they had conducted an action plan, with a (free-description) question: “What kind of action plan did you make at the start of health promotion practice?”

### Data analysis

The primary outcomes (changes in physical activity and healthy eating) were confirmed via a linear mixed model. Effect sizes (*R*^*2*^) were calculated to identify the effectiveness of participants’ current practice [32]. Effect sizes were assessed as 0.01 = small, 0.09 = medium, and 0.25 = large [33]. Statistical analyses of quantitative data were conducted with SPSS version 24 (IBM Japan, Tokyo, Japan) and R version 3.3.1.

For the secondary outcome analysis, self-reported positive change following the manga trial were coded and summarized using a thematic analysis process [34]. Thematic analysis is a well-established qualitative method to understand a psychological phenomenon. Retrieved qualitative data were analyzed in four processes: (1) reviewing and becoming familiar with the qualitative data provided by the participants; (2) identifying codes and assigning the relevant description for each code; (3) considering the themes from classifying codes; and (4) reviewing and reconfirming the themes. Construction of a thematic map was not possible in this study because qualitative descriptive data were insufficient to constrict a hypothetical model. Specific action plans retrieved from responses to the free-description question were also summarized according to the thematic analysis.

## Results

### Participants’ characteristics

Table 3 shows participants’ demographic characteristics. In total, 67% of participants were female (*n* = 8). Participants’ mean age was 62.1 years (standard deviation [*SD*] = 7.7), with a range of 47–73 years. Three participants were employed full-time, two were part-time employees, six were housewives, and one was unemployed. All participants set specific action plans involving one to three actions. An equal number of participants set physical activity plans and healthy eating plans (both *n* = 7). Three participants planned to improve both physical activity and eating behavior. Only one participant focused on body weight (i.e., planned to monitor their own weight).

**Table 3:** Participants’ characteristics

No.	Gender	Age (years)	Job	Action plan		
				Physical activity	Eating behavior	Other
1	Female	50s	Part-time employee	Proactively going for walks		
2	Female	60s	Housewife	Standing on the train		
3	Female	60s	Housewife	Yoga	Restrict alcohol intake; Eat vegetables first; Eat moderately	
4	Female	60s	Housewife	Proactively going for walks		
5	Female	50s	Housewife	Radio gymnastic exercises		
6	Male	60s	Farmer			Monitor weight
7	Male	70s	Carrier business		Carry a packed lunch; Intake of vegetables	
8	Female	40s	Part-time employee	Uses a pedometer	Eat vegetables first	
9	Female	70s	Housewife		Stretch exercises	
10	Female	50s	Housewife	Radio gymnastic exercises	Chew well while eating	
11	Male	60s	Unemployed		Restrict carbohydrates intake	
12	Male	60s	Self-employed		Restrict salt intake	

### Changes in primary outcomes

As shown in Table 4, there were no significant positive changes in physical activity. Slight changes in vigorous, moderate, and moderate-vigorous physical activity scores were observed with small effect sizes (*R*^*2*^ = 0.02, 0.01, and 0.02, respectively), although these changes did not reach statistical significance.

**Table 4:** Changes in physical activity and eating behavior

	Baseline		1 month		Estimate					
	*Mean*	(*SE*)	*Mean*	(*SE*)	Intercept	(95% CI)	Time	(95% CI)	*p-value*	*R* ^*2*^
*IPAQ*										
Vigorous PA	285	(205.5)	428.3	(205.5)	428.3	(1.9, 854.8)	−143.3	(− 754.2, 467.5)	0.62	0.02
Moderate PA	590.8	(230.5)	617.5	(230.5)	617.5	(134.4, 1100.6)	−26.7	(− 570.8, 517.4)	0.92	0.01
MVPA	875.8	(393.8)	942.0	(409.3)	942.0	(87.0, 1797.1)	−66.2	(−10.90.3, 957.9)	0.89	0.02
Walking	332.5	(120.9)	187.5	(122.6)	187.5	(−76.3, 451.4)	145.0	(−15.1, 305.0)	0.07	N.A.
Sedentary (week days)	205	(33.4)	207.5	(33.4)	207.5	(137.2, 277.8)	−2.5	(−74.5, 69.5)	0.94	N.A.
Sedentary (holidays)	239.4	(37.1)	260	(36.5)	260	(−75.6, 34.4)	−20.6	(−75.6, 34.4)	0.43	N.A.
*HEBI-J*										
Balance	15.1	−0.8	15.5	−0.8	15.5	(13.7, 17.3)	−0.4	(−1.3, 0.5)	0.32	0.09
Pattern	15.1	−0.8	16	−0.8	16.0	(14.3, 17.7)	−0.9	(−2.5, 0.6)	0.21	0.16
Restriction	13.3	−0.8	14.3	−0.8	14.3	(12.6, 15.9)	−1	(−2.1, 0.2)	0.1	0.25
Total	43.2	−2.2	46.2	−2.2	46.2	(41.5, 50.9)	−3.1	(−5.6, −0.6)	0.02*	0.47

*p < .01. Notes. *CI* confidence interval, *SE* standard error, *PA* physical activity, *MVPA* moderate-vigorous physical activity, Physical activity scores calculated by minutes per week, *IPAQ* International Physical Activity Questionnaire, *HEBI-J* Healthy Eating Behavior Index for Japanese, *N.A.* of effect size (*R*^*2*^) means effect size calculation was not available because of physical activity scores were decreasing from baseline to one month; Criteria for effect sizes (*R*^*2*^): > 0.01 = small, > 0.09 = medium, and > 0.25 = large (Cohen, 1988)

For healthy eating behaviors, total healthy eating scores showed a significant increase (*p* < 0.05, *R*^*2*^ = 0.47). Medium effect sizes were observed for Balance (*R*^*2*^ = 0.09) and Pattern (*R*^*2*^ = 0.16), although the differences were not significant.

### Secondary outcomes

Psychological changes were observed for nine participants (age: *M* = 61.33, *SD* = 7.67; gender: male = 3, female = 6). Table 5 displays the thematic analysis. Eight codes and three themes were identified. Theme 1 reflected “strengthening the antecedent factors of actions” (improvement in intention of carrying out actions, recognizing and understanding the importance of actions, awareness of the importance of changes in actions, and enhanced self-efficacy for carrying out actions). Theme 2 represented “the benefits of using manga or comic strips” (content comprehension aided by pictures, sensory understanding). Theme 3 encompassed “requests for information” (information on calories burnt and specifying expectations regarding results using numbers).

**Table 5:** Participants’ feedback on the manga health promotion intervention

Theme	Code	Description example
Benefits of using manga or comic strips	Content comprehension aided by pictures	Since it is comic strip format it is easy to understand and I think it is good to understand using pictures with less language used. (No. 9)
	Sensory understanding	I could understand what to do in one glance. (No. 10)
Request for information	Information on calories burnt	It would be helpful to have the average value of calories burnt (No. 6)
	Specify expectations regarding results using numbers	I think it is good to know the extent of the difference between when one does small things diligently and when one does not in the form of numbers. (No. 8)
Strengthening the antecedent factors of actions	Improvement in the intention of carrying out actions	On some occasions I made it a point not to sit in the train and I would like to continue this (No. 4).
	Recognition and understanding of the importance of actions	I reconfirm things that I was thinking of doing by looking at the pictures and then practice them. (No. 9)
	Awareness of the importance of changes in actions	I thought that I could also do such things and doing them would make a difference (No. 8)
	Enhancing self-efficacy for carrying out actions	I think I can do “simultaneous” exercises, watch my calories, and eat vegetables and fruits instead of sweets (No. 11)

Note. Numbers in parentheses denote participant number

## Discussion

This case study aimed to confirm the feasibility of a manga intervention to promote physical activity and healthy eating. The analysis of primary outcomes showed a significant positive change in healthy eating behaviors. However, physical activity showed negative changes post-test. A previous meta-analysis of behavior change interventions for physical activity and nutrition education reported equal effect sizes [35]. Season bias and a ceiling effect might explain why there was no change or decrease in physical activity. The present intervention was conducted in July, according to the circumstances of the intervention community. In July, temperatures soared to 38–39 °C in the intervention area [36]. Previous research indicated general physical activity levels increased from spring to summertime (April to August), although high temperature resulted in decreased physical activity [37]. In addition, five participants exceeded the World Health Organization recommendation of moderate-vigorous physical activity for 150 min per day [38] at baseline (i.e., a ceiling effect was observed). Participants in this study seemed to show high interest in health promotion and engaging in daily physical activity, although they had metabolic syndrome. Therefore, these results indicate the primary risk factor in the present participants was unhealthy eating behavior.

The secondary outcome analysis indicated that using manga contributed to improving awareness, understanding of the importance of health promotion, and increasing self-efficacy and intention to change unhealthy behaviors. These findings are consistent with previous qualitative research. Previous study reported psychological advantages of using health promotion manga, including a sense of resonance, reassurance, empathy, companionship, and captivation [39]. Although the positive impact of health promotion manga on actual behavior change was limited, manga interventions appear to have a positive effect on psychological readiness.

This study had several limitations. First, recruitment was conducted on a voluntary basis. Participants had already achieved guideline recommendations for physical activity. Therefore, selection bias might have influenced the results. Second, seasonal bias might have influenced the study results. Future interventions should avoid the summer season. Third, the present study only involved a 1-month trial. Therefore, we did not consider long-term effects. Fourth, previous studies demonstrated the existence of a psychological mediator in engaging in health behavior in entertainment education, such as identification, reassurance, and self-awareness [6]. The present study also observed psychological variables, such as comprehension based on graphic effects and sensory understanding. However, we did not consider the mediator effect of psychological variables in engaging in health behavior.

## Conclusions

Despite several limitations, the present findings contribute to facilitating the use of manga in health promotion in Japan. Further study is needed to identify psychological mediators and their effect in manga health promotion interventions [14]. In addition, randomized control trials are needed to confirm the impact of health promotion manga on health behavior change and health outcomes [10].

Few studies have reported actual effects of manga interventions in patient education [10]. Further randomized controlled trials, systematic reviews, and meta-analyses are necessary to demonstrate the advantage of manga compared with existing treatment.

In the practical healthcare setting, financial support to produce manga is a barrier to manga-based interventions [40]. Cooperation of university/research institute researchers and health practitioners is necessary. In addition, recruitment of participants with low health literacy or low intention to change their unhealthy lifestyle is a serious concern. Using incentives and providing follow-up or tailored programs may increase the recruitment rate and adherence to interventions [41].

## Additional files

Additional file 1: Health promotion manga. (PDF 3263 kb)

Additional file 2: Personalized letter. (PDF 2462 kb)

Additional file 3: Feedback messages. (PDF 6265 kb)

## Abbreviations

AGFI: Adjusted Goodness-of-Fit Index
CFI: Comparative Fit Index
CI: Confidence Interval
GFI: Goodness-of-Fit Index
HDL: High Density Lipoprotein
HEBI-J: Healthy Eating Behavior Index for Japanese
IPAQ: International Physical Activity Questionnaire
MVPA: Moderate-Vigorous Physical Activity; Physical activity scores calculated by minutes per week
PA: Physical Activity
RMSEA: Root Mean Square Error of Approximation
SE: Standard Error
SRMR: Standardized Root Mean Square Residual

## References

[1] World Health Organization: Noncommunicable diseases. http://www.who.int/mediacentre/factsheets/fs355/en/ (2015). Accessed 9 Jan 2018.

[2] Japanese Ministry of Health, Labour and Welfare: National Health and Nutrition Survey (In Japanese). http://www.mhlw.go.jp/stf/houdou/0000032074.html (2012). Accessed 9 Jan 2018.

[3] Jensen MD, Ryan DH, Apovian CM, Ard JD, Comuzzie AG, Donato KA (2014). AHA/ACC/TOS guideline for the management of overweight and obesity in adults: a report of the American College of Cardiology/American Heart Association task force on practice guidelines and the Obesity Society. Circulation.

[4] World Health Organization: Diet and physical activity: A public health priority (Global Strategy on Diet, Physical Activity and Health). http://www.who.int/dietphysicalactivity/background/en/ (2014). Accessed 9 Jan 2018.

[5] Fletcher GM, Behrens TK, Domina L (2008). Barriers and enabling factors for work-site physical activity programs: a qualitative examination. J Phys Act Health.

[6] Literat I, Chen NTN (2014). Communication infrastructure theory and entertainment-education: an integrative model for health communication. Communic Theor.

[7] Reinermann JL, Lubjuhn S, Bouman M, Singhal A (2014). Entertainment-education: storytelling for the greater, greener good. Int J Sust Dev.

[8] Singhal A, Wang H, Roger EM, Rice RE, Atkin CK (2013). The rising tide of entertainment-education in communication campaign. Public communication campaigns (4th ed.).

[9] Volk RJ, jibaja-Weiss ML, Hawley ST, Kneuper S, Spann SJ, Miles BJ, et al. Entertainment education for prostate cancer screening: a randomized trial among primary care patients with low health literacy. Patient Educ Couns. 2008;73:482–9. 10.1016/j.pec.2008.07.033 PMC2867348 18760888

[10] Branscum P, Sharma M (2009). Comic books an untapped medium for health promotion. Am J Health Stud.

[11] Dworkin MS, Peterson CE, Gao W, Mayor A, Hunter R, Negron E (2013). Efficacy of a food safety comic book on knowledge and self-reported behavior for persons living with AIDS. PLoS One.

[12] Bandura A (2001). Social cognitive theory: an agentic perspective. Annu Rev Psychol.

[13] Dahlstrom MF (2014). Using narratives and storytelling to communicate science with nonexpert audiences. Proc Natl Acad Sci U S A.

[14] Leung MM, Tripicchio G, Agaronov A, Hou N (2014). Manga comic influences snack selection in black and Hispanic new York City youth. J Nutr Educ Behav.

[15] Houts PS, Doak CC, Doak LG, Loscalzo MJ (2006). The role of pictures in improving health communication: a review of research on attention, comprehension, recall, and adherence. Patient Educ Couns.

[16] Daliot-Bul M (2009). Japan brand strategy: the taming of ‘cool Japan’ and the challenges of cultural planning in a postmodern age. Soc Sci Jpn J.

[17] Ito K (2005). A history of manga in the context of Japanese culture and society. J Pop Cult.

[18] Shimazaki T, Maeba K, Takenaka K. Assessment of citywide health promotion campaign using cross-sectional study method: a case report from a Japanese suburb community. SAGE Research Methods Cases 2017. http://methods.sagepub.com/case/citywide-health-promotion-cross-sectional-study-japanese-suburb-community (accessed 9 January 2018).

[19] Ministry of Health, Labour and Welfare: Annual health, labour and welfare report 2008–2009. http://www.mhlw.go.jp/english/wp/wp-hw3/02.html (2010). Accessed 9 Jan 2018.

[20] Borovoy A (2017). Japan’s public health paradigm: governmentality and the containment of harmful behavior. Med Anthropol.

[21] Ministry of Health, Labour and Welfare: Policy reports: specific health checkup (metabolic syndrome checkup) and specific health guidance (In Japanese). http://www.mhlw.go.jp/seisaku/2009/09/02.html (2009). Accessed 9 Jan 2018.

[22] Japan Health Insurance Association: Criteria of specific health checkup (In Japanese). https://www.kyoukaikenpo.or.jp/g4/cat410/sb4020/ra104 (2016). Accessed 9 Jan 2018.

[23] Hill JO, Peters JC, Wyatt HR (2009). Using the energy gap to address obesity: a commentary. J Am Diet Assoc.

[24] Lutes LD, Steinbaugh EK (2011). Theoretical models for pedometer use in physical activity interventions. Phys Ther Rev.

[25] Shimazaki T, Iio M, Lee YH, Konuma K, Takenaka K (2017). Exploring physical activity with a low psychological burden and high feasibility in Japan: a qualitative study. Psychol Health Med.

[26] Shimazaki T, Iio M, Lee YH, Suzuki A, Konuma K, Teshima Y (2016). Construction of a short form of the healthy eating behaviour inventory for the Japanese population. Obes Res Clin Pract.

[27] Loprinzi PD, Cardinal BJ (2013). Association between biologic outcomes and objective measured physical activity accumulated in >10-minite bouts and <10-minute bouts. Am J Health Promot.

[28] Lutes LD, Daiss SR, Barger SD, Read M, Steinbaugh E, Winett RA (2012). Small changes approach promotes initial and continued weight loss with a phone-based follow-up: nine-month outcomes from ASPIRES II. Am J Health Promot.

[29] Craig CL, Marshall AL, Sjöström M, Bauman AE, Booth ML, Ainsworth BE (2003). International physical activity questionnaire: 12-country reliability and validity. Med Sci Sport Exer.

[30] Murase N, Katsumura T, Ueda C, Inoue S, Shimomitsu T (2002). Validity and reliability of Japanese version of international physical activity questionnaire. Journal of Health and Welfare Statistics.

[31] Kristal AR, Glanz K, Curry SJ, Patterson RE (1999). How can stages of change be best used in dietary interventions?. J Am Diet Assoc.

[32] Field A (2015). Discovering statistics using IBM SPSS statistics.

[33] Cohen J (1988). Statistical power analysis for the behavioral sciences.

[34] Braun V, Clarke V (2006). Using thematic analysis in psychology. Qual Res Psychol.

[35] Michie S, Abraham C, Whittington C, McAteer J (2009). Effective techniques in healthy eating and physical activity interventions: a meta-regression. Health Psychol.

[36] Japan Meteorological Agency: Weather data searching (In Japanese). http://www.data.jma.go.jp/obd/stats/etrn/index.php (2017). Accessed 9 Jan 2018.

[37] Tucker P, Gilliland J (2007). The effect of season and weather on physical activity: a systematic review. Public Health.

[38] World Health Organization: Global recommendations on physical activity for health. http://www.who.int/dietphysicalactivity/publications/9789241599979/en/ (2010). Accessed 9 Jan 2018. 26180873

[39] McNicol S (2017). The potential of educational comics as a health information medium. Health Inf Libr J.

[40] Fossard E, Lande R (2008). Entertainment-education for better health. INFO Report (Johns Hopkins Bloomberg School of Public Health, Center for Communication Programs, The INFO Project).

[41] Edwards P, Roberts I, Clarke M, DiGuiseppi C, Pratap S, Wentz R, et al. Increasing response rates to postal questionnaires: systematic review. BMJ. 2002;324:1183–5. 10.1136/bmj.324.7347.1183 PMC111107 12016181

