# Supplementary material for: Use of health promotion manga to encourage physical activity and healthy eating in Japanese patients with metabolic syndrome: a case study
Source: Arch Public Health. 2018 Jun 18;76:26. doi: 10.1186/s13690-018-0273-5 (PMC6004677; doi:10.1186/s13690-018-0273-5)
Supplement: Supplementary file 1 — Health promotion manga. (PDF 3263 kb) [file 13690_2018_273_MOESM1_ESM.pdf]

わずかな努力が大きな成果に！

スモールチェンジをはじめよう！

Small Change スモールチェンジ

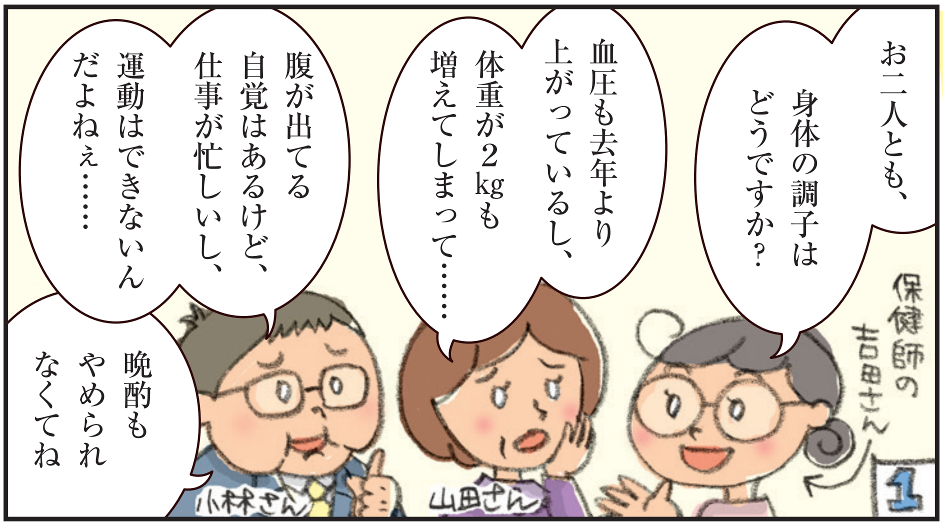

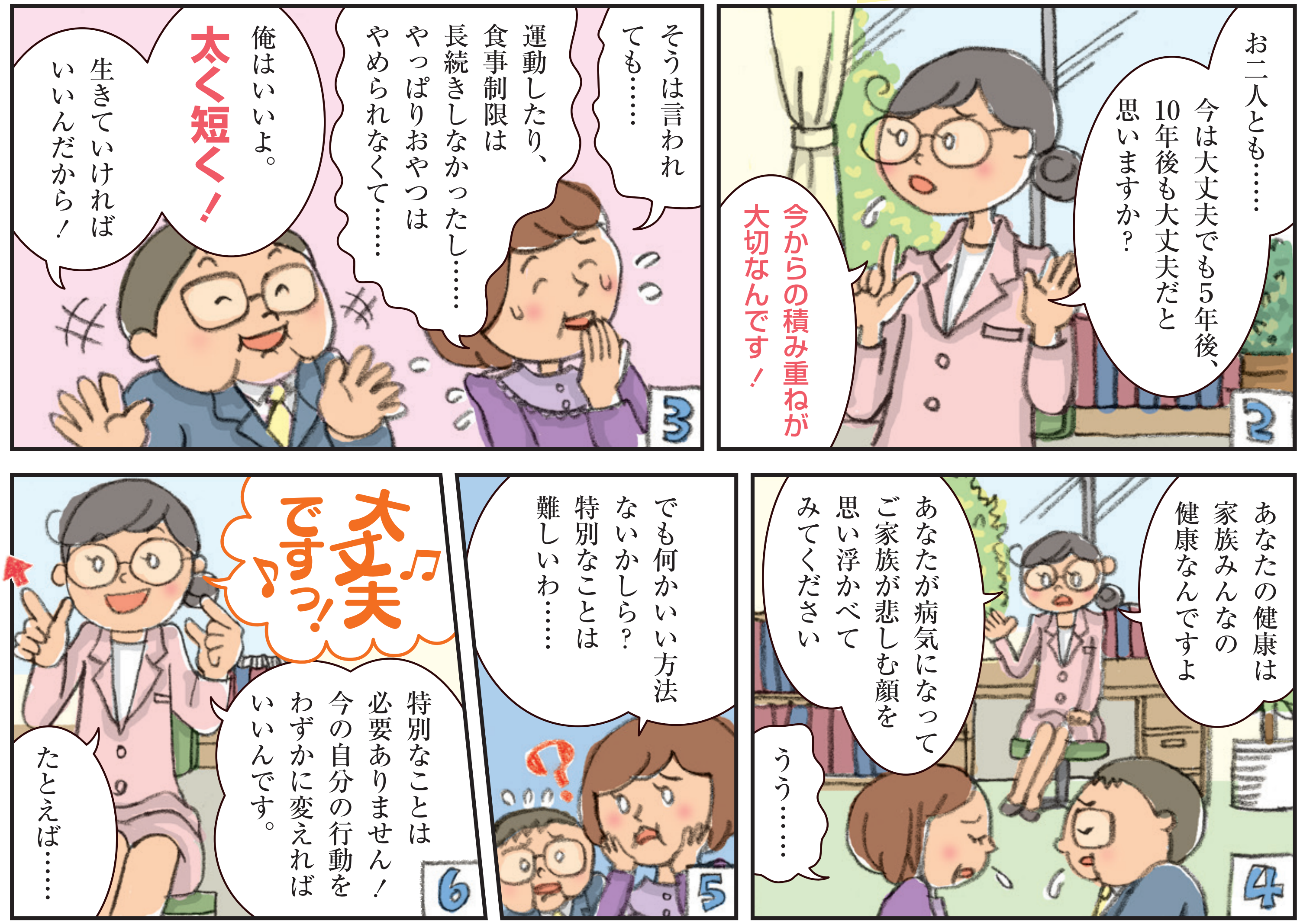

はじめよう！スモールチェンジ

こまぎれウォーキングも効果あり！

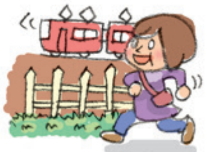

ウォーキングというと、シューズを履いてトレーニングウェアを着て、一度に長い時間続けないと効果が出ないように感じる方も多いはず……でも最近の研究では、一回の時間は長くても短くても効果は一緒で、こまぎれでもたくさん歩くことが大切だというのがわかってきました。忙しくて運動ができない方でも、通勤、買い物、お出かけの「ちょっとした移動を歩きに変える」、スモールチェンジから始めてみませんか？

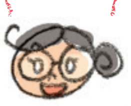

質・量・頻度、行いやすいところから！

スモールチェンジといっても何をしたら良いのか思い浮かばない。そんな方は、小林さんや山田さんのように今行っている行動の「質を変える・量を変える・頻度を変える」という3つの面から考えてみましょう。自分にあったスモールチェンジ目標が見つかります。

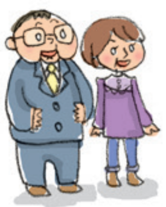

わずかにできる健康づくり「スモールチェンジ活動」に関する情報が満載！
埼玉県ときがわまちスモールチェンジウェブサイト
http://www.town.tokigawa.lg.jp/forms/info/info.aspx?info_id=27426

作成：早稲田大学応用健康科学研究室
研究助成：笹川スポーツ財団 SASAKAWA SPORTS FOUNDATION

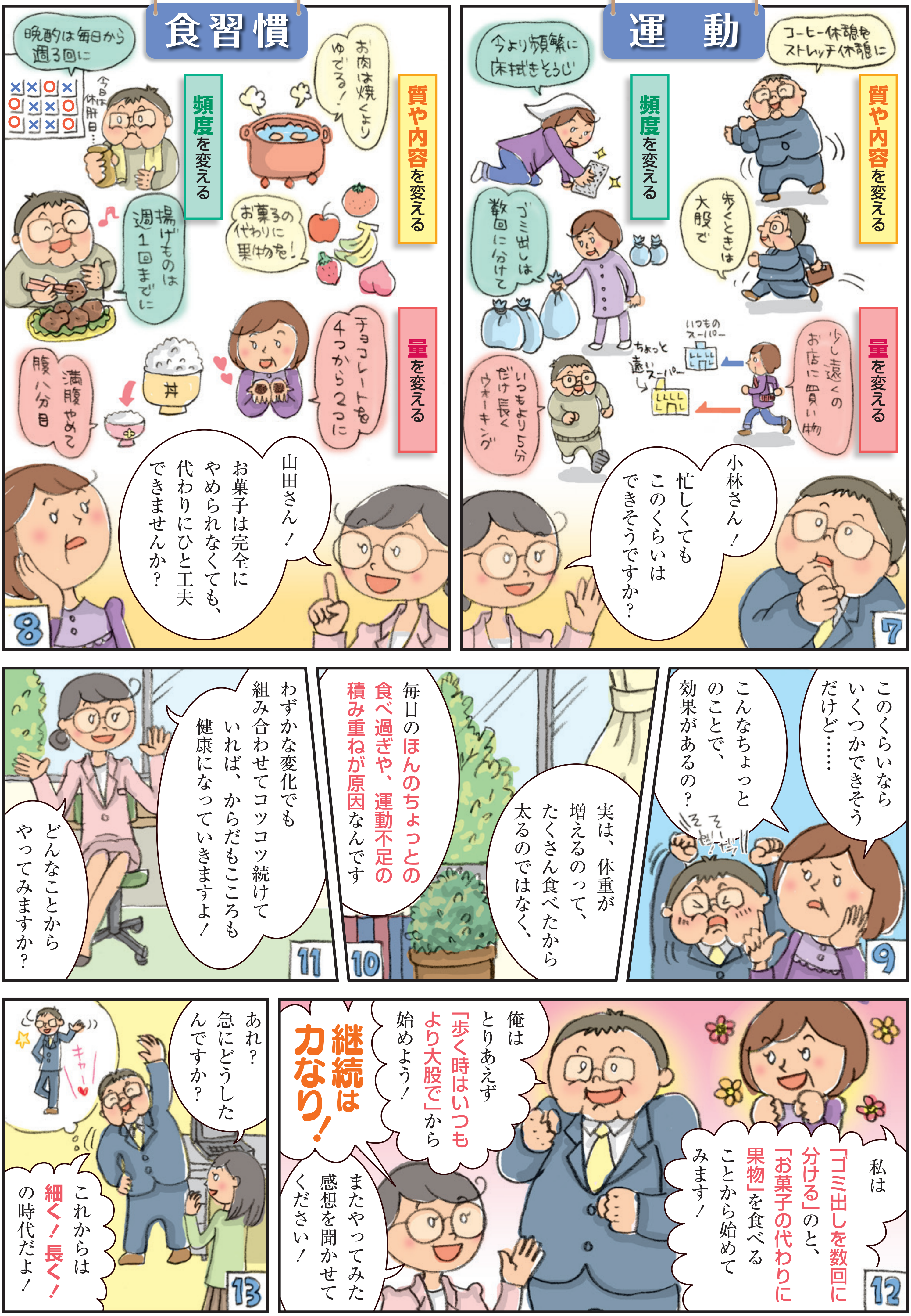

運　動
頻度を変える
今より頻繁に床拭きそうじ
ゴミ出しは数回に分けて
質や内容を変える
コーヒー休憩をストレッチ休憩に
歩くときは大股で
量を変える
少し遠くのお店に買い物
いつものスーパー
ちょっと遠いスーパー
いつもより5分だけ長くウォーキング
小林さん！
忙しくても
このくらいは
できそうですか？
食習慣
頻度を変える
晩酌は毎日から週3回に
今日は休肝日…
揚げものは週1回までに
質や内容を変える
お肉は焼くよりゆでる！
お菓子の代わりに果物を！
量を変える
チョコレートを4つから2つに
満腹やめて腹八分目
丼
山田さん！
お菓子は完全に
やめられなくても、
代わりにひと工夫
できませんか？
このくらいならいくつかできそうだけど……
こんなちょっとのことで、効果があるの？
実は、体重が増えるのって、たくさん食べたから太るのではなく、
毎日のほんのちょっとの食べ過ぎや、運動不足の積み重ねが原因なんです
わずかな変化でも組み合わせてコツコツ続けていれば、からだもこころも健康になっていきますよ！
どんなことからやってみますか？
私は「ゴミ出しを数回に分ける」のと、「お菓子の代わりに果物」を食べることから始めてみます！
俺はとりあえず「歩く時はいつもより大股で」から始めよう！
継続は力なり！
またやってみた感想を聞かせてください！
あれ？急にどうしたんですか？
キャー
これからは細く！長く！の時代だよ！
