# Supplementary material for: Use of health promotion manga to encourage physical activity and healthy eating in Japanese patients with metabolic syndrome: a case study
Source: Arch Public Health. 2018 Jun 18;76:26. doi: 10.1186/s13690-018-0273-5 (PMC6004677; doi:10.1186/s13690-018-0273-5)
Supplement: Supplementary file 2 — Personalized letter. (PDF 2462 kb) [file 13690_2018_273_MOESM2_ESM.pdf]

English Version

(Participant's Name)
Current Condition to Engage Health Promotion Behavior

Changing Physical Activity

Day per week (day or days)

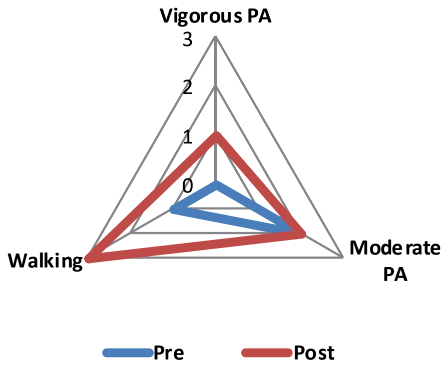

Minutes per day (min)

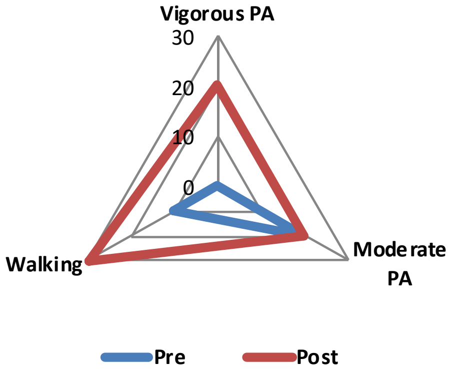

Changing Eating Behavior

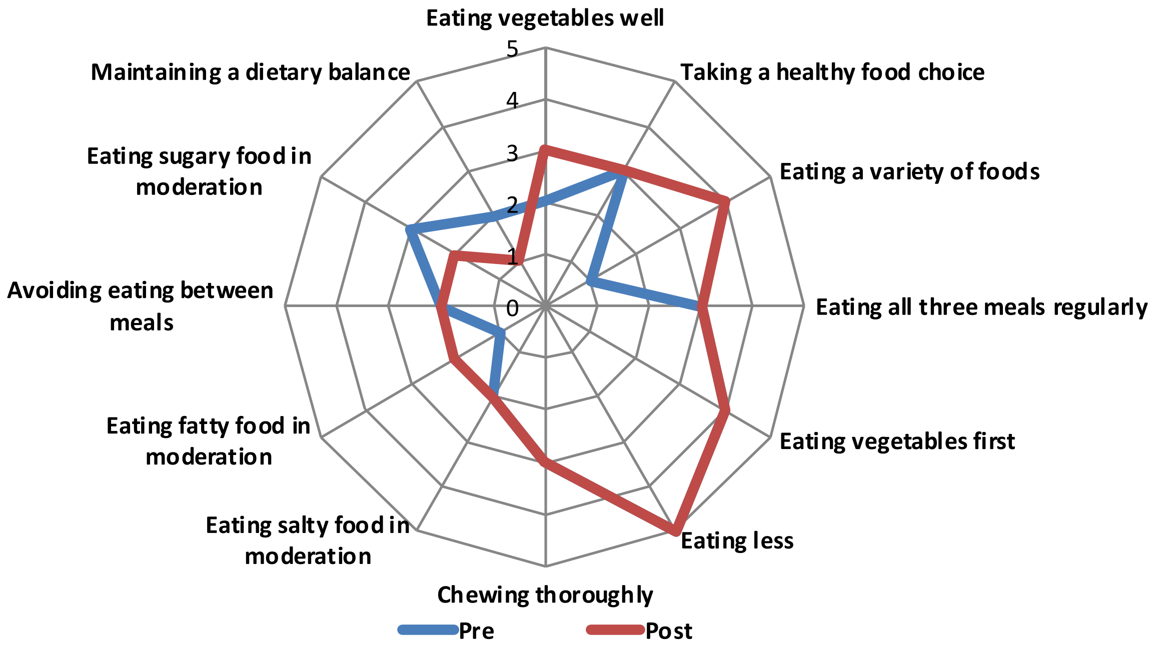

Tips for Becoming More Healthy

Be careful to excessive consumption of sugar in water replacement

Water replacement is important not only sweating during exercise but also daily living. In addition, what do you drink is also important for weight control. For example, there are three table spoons of sugar was contained in 500 ml of sports supplement drink. Sometimes, water replacement touch overweight or obesity. Drinking dilute by adding water of sports supplement drink or changing to drink tea also contribute to prevent obesity or overweight.

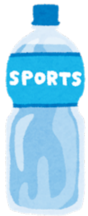

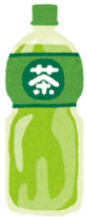

Physical Activity

Don’t let the weather beat you! Enjoy the Tokigawa autumn!

Autumn brings a break from the summer heat. Making an effort to go for a walk or do some exercise is tough. Lots of people just give up. This is now a good time of year for us to get some exercise. Walking or exercising with our friends is a great way to give our minds and bodies a workout while enjoying nature in Tokigawa. Don’t forget you might have to cope with the rain. Have a few exercises and simple muscle toning routines ready that you can do at home.

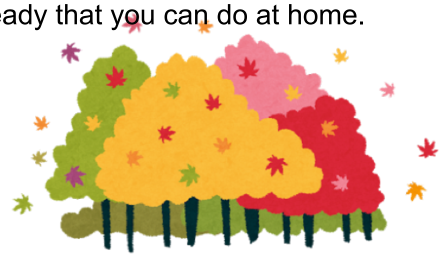
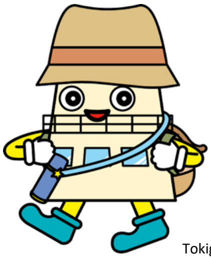
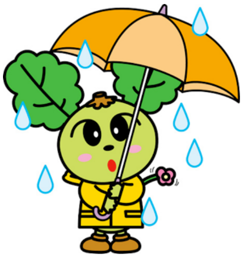

Tokigawa character mascot: Doma-kun and Norabitan

Eating Behavior

A tip for making miso soup!
Add a lot of fresh vegetables.

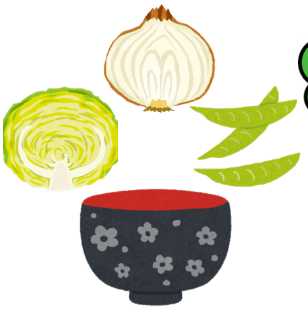
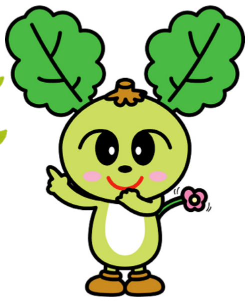

Tokigawa character mascot: Norabitan

Think about which of these you don’t eat very much: carbohydrates, proteins, lipids, vitamins, and minerals

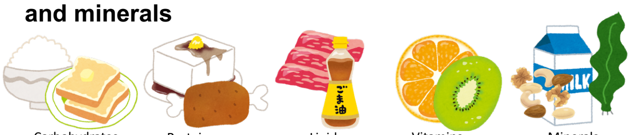

Carbohydrates Protein Lipids Vitamins Minerals

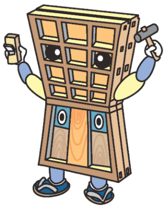

Tokigawa character mascot: Shoji-kun

Japanese Version

（対象者名）さんの健康づくり行動の状況

身体活動の実施状況の変化

週あたりの日数（日）

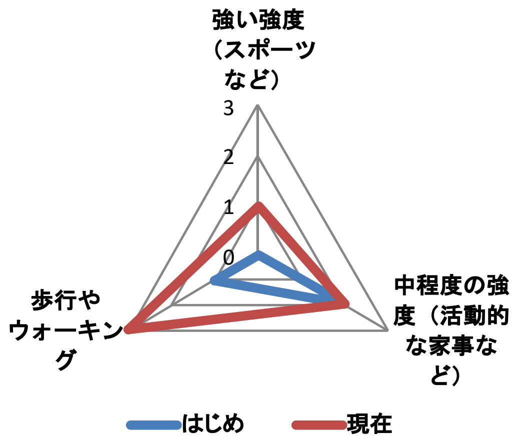

一日あたりの時間（分）

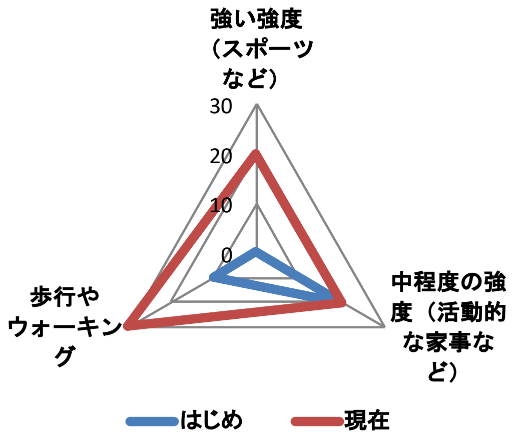

食習慣の変化

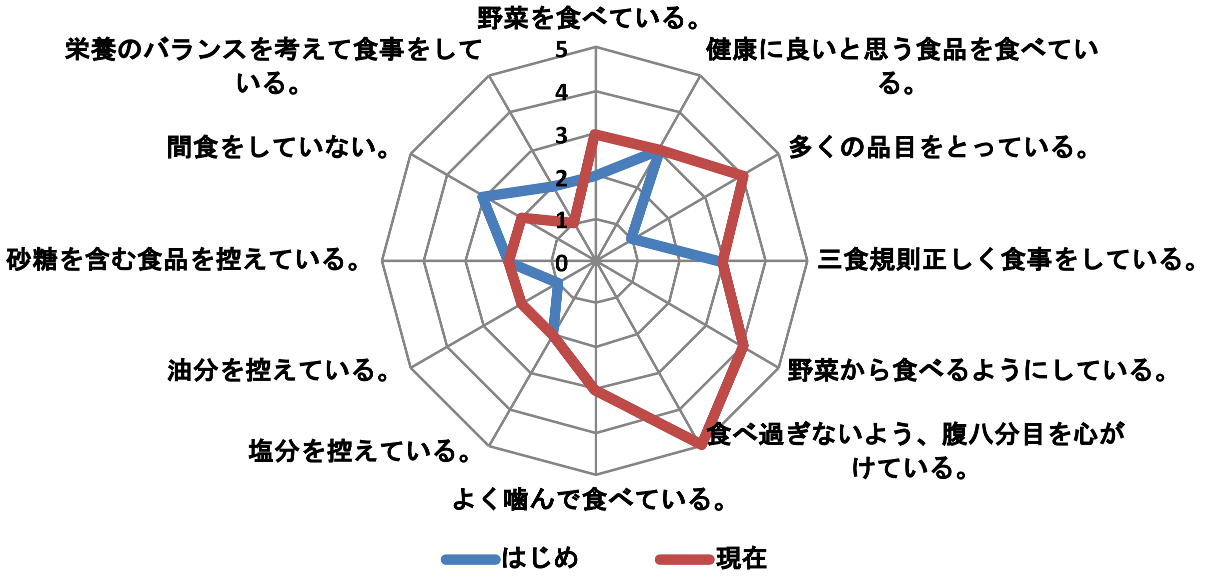

あなたにおすすめの健康づくり

【水分補給にひそむ！砂糖のとり過ぎにご用心！】

しっかり汗をかいて運動した時も，日常生活でも水分補給は大切です。ですが，何を飲むかも大切です。例えば，スポーツドリンクは，500ml ペットボトルひとつで約大さじ3杯もの糖分含まれています。水分補給を心がけて太ってしまう方も少なくありません。

スポーツドリンクを水で薄めて飲む，お茶に変えるこれだけでも肥満の予防になります。

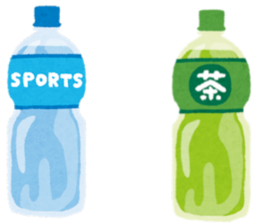

身体活動

【天気に負けず！ときがわの秋を楽しみましょう！】

暑い夏をはさみ，せっかく意識して行っていたウォーキングや体操もつらくなり，やめてしまった方も多いはず。これから身体を動かすのにもよい季節になっていきます。友人とのウォーキングや体操，ときがわの自然を楽しみながら心も身体も活動的に。また，雨への対処も忘れずに。家でできる体操や簡単な筋トレを準備しておきましょう。

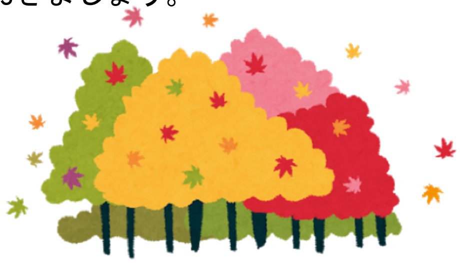

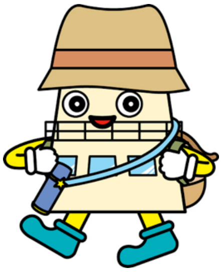

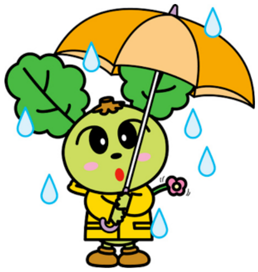

ときがわ町マスコットキャラクター:ドームくんとのラビたん

食事

お味噌汁に一工夫！
たくさん野菜を入れましょう。

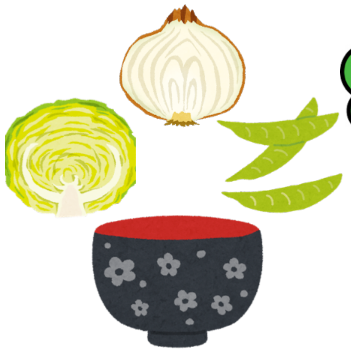

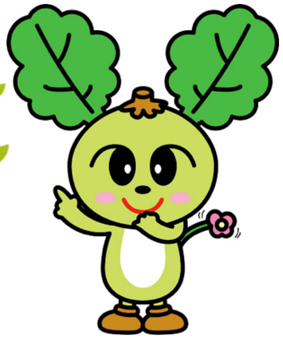

ときがわ町マスコットキャラクター:のラビたん

炭水化物，タンパク質，脂質，ビタミン，ミネラル，あまり食べないものはどれか考えてみましょう。

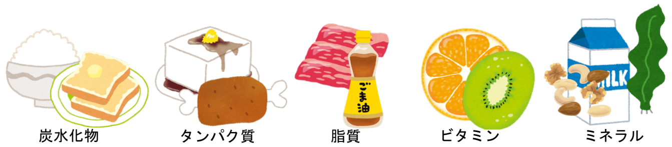

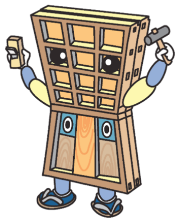

ときがわ町マスコットキャラクター:しょうじくん
