# Supplementary material for: Use of health promotion manga to encourage physical activity and healthy eating in Japanese patients with metabolic syndrome: a case study
Source: Arch Public Health. 2018 Jun 18;76:26. doi: 10.1186/s13690-018-0273-5 (PMC6004677; doi:10.1186/s13690-018-0273-5)
Supplement: Supplementary file 3 — Feedback messages. (PDF 6265 kb) [file 13690_2018_273_MOESM3_ESM.pdf]

English Version

Feedback Messages

Personalized message was selected according to change in physical activity and eating behavior during the intervention period .

Physical Activity 1: For decreased participants

Quality, quantity, frequency: Make a small adjustment and do a little bit more than before.

Everyone has tried hard to exercise during the hot summer. Autumn is a good time of year
to step it up a little. The benefits of what we have done thus far have accumulated. Just a small change; a little bit more than before is good for the mind and the body! Think about your everyday routine and try to make a small adjustment, as shown below, to the quality, quantity, or frequency of what you do – something you can easily incorporate into your routine.

Walk

Walk	Change the Quality (Content)	Think about walking more briskly
	Change the Quantity	10-15 mins
	Change the Frequency	Do this twice a week instead of once a week

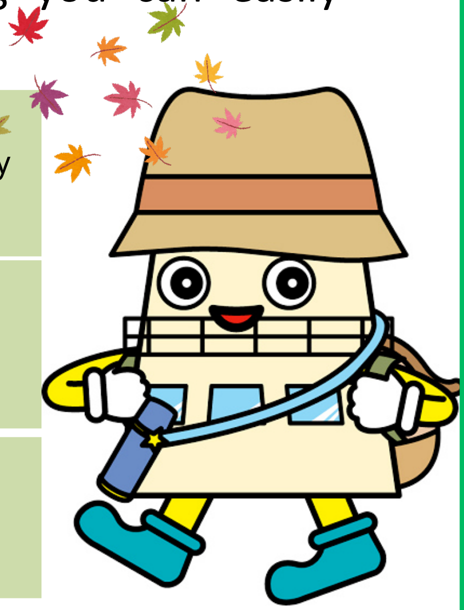

Tokigawa character mascot: Doma-kun

Physical Activity 2: For increased participants

Don’t let the weather beat you! Enjoy the Tokigawa autumn!

Autumn brings a break from the summer heat. Making an effort to go for a walk or do some exercise is tough. Lots of people just give up. This is now a good time of year for us to get some exercise. Walking or exercising with our friends is a great way to give our minds and bodies a workout while enjoying nature in Tokigawa. Don’t forget you might have to cope with the rain. Have a few exercises and simple muscle toning routines ready that you can do at home.

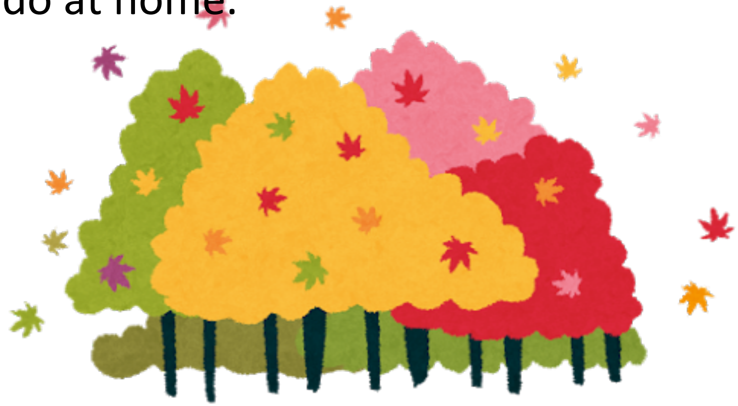

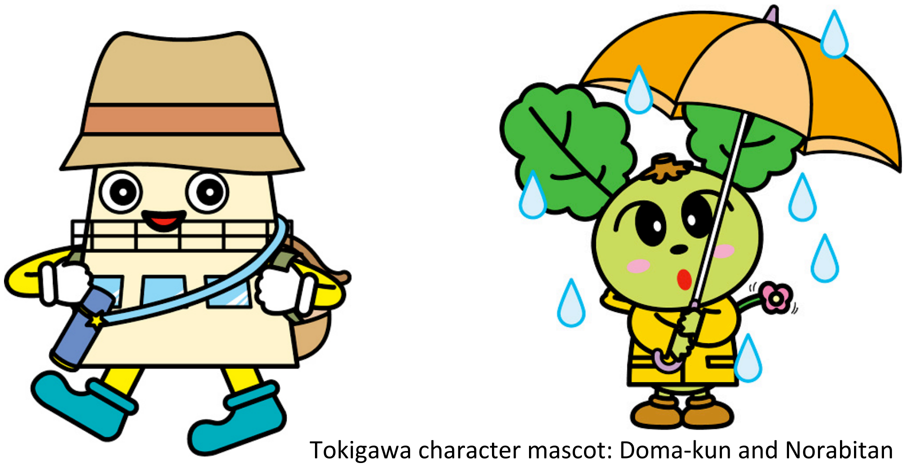

Tokigawa character mascot: Doma-kun and Norabitan

Eating Behavior 1:Promote eating vegetables well

A tip for making miso soup!
Add a lot of fresh vegetables.

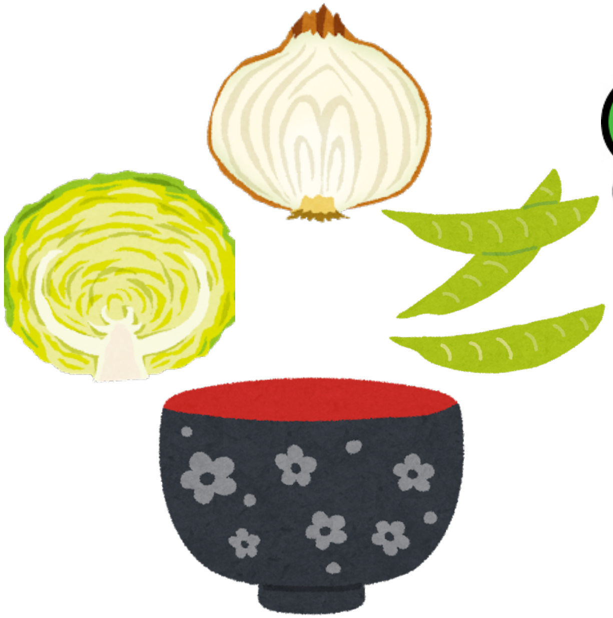

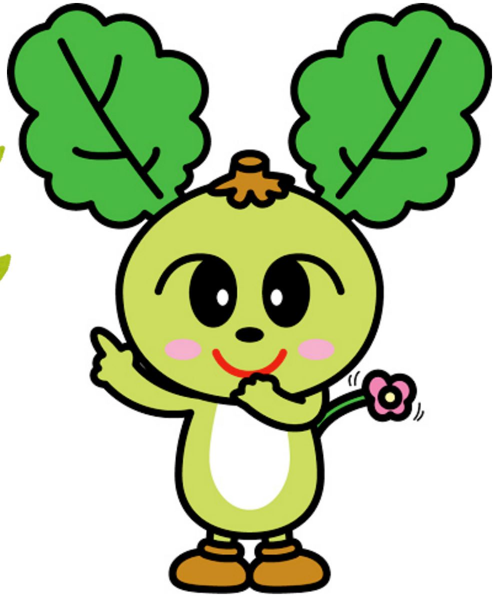

Tokigawa character mascot: Norabitan

Eating Behavior 2: Promote taking a healthy food choice

First of all, let’s eat food that’s good for us – such as yogurt or blueback fish.

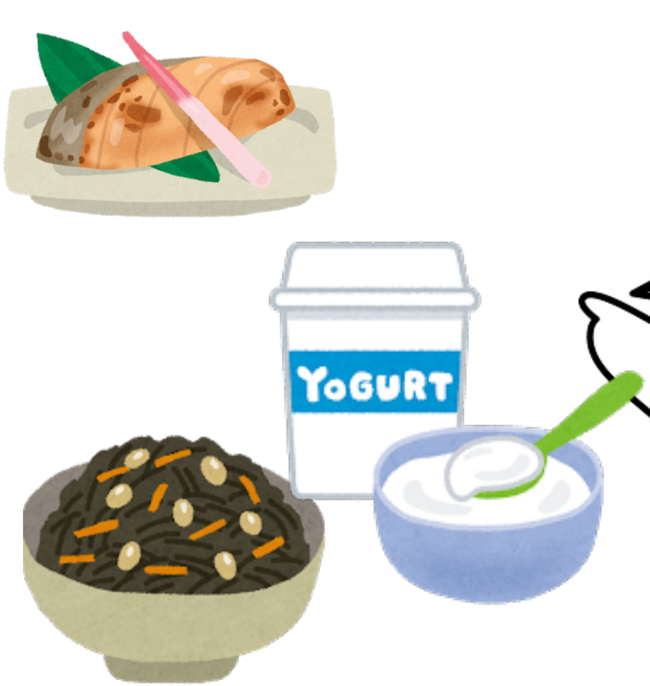

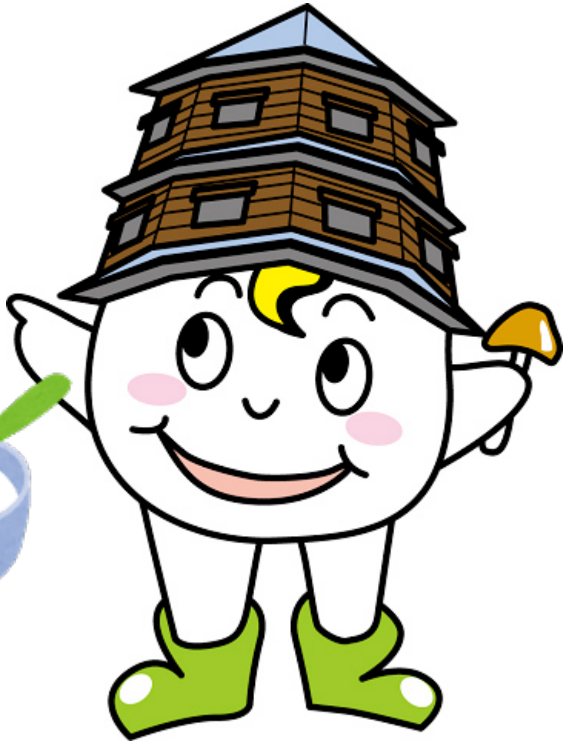

Tokigawa character mascot: Ono-kun

Eating Behavior 3: Eating a variety of foods

Steam a lot of ingredients together!
Even if you have a small appetite, you can easily eat plenty.

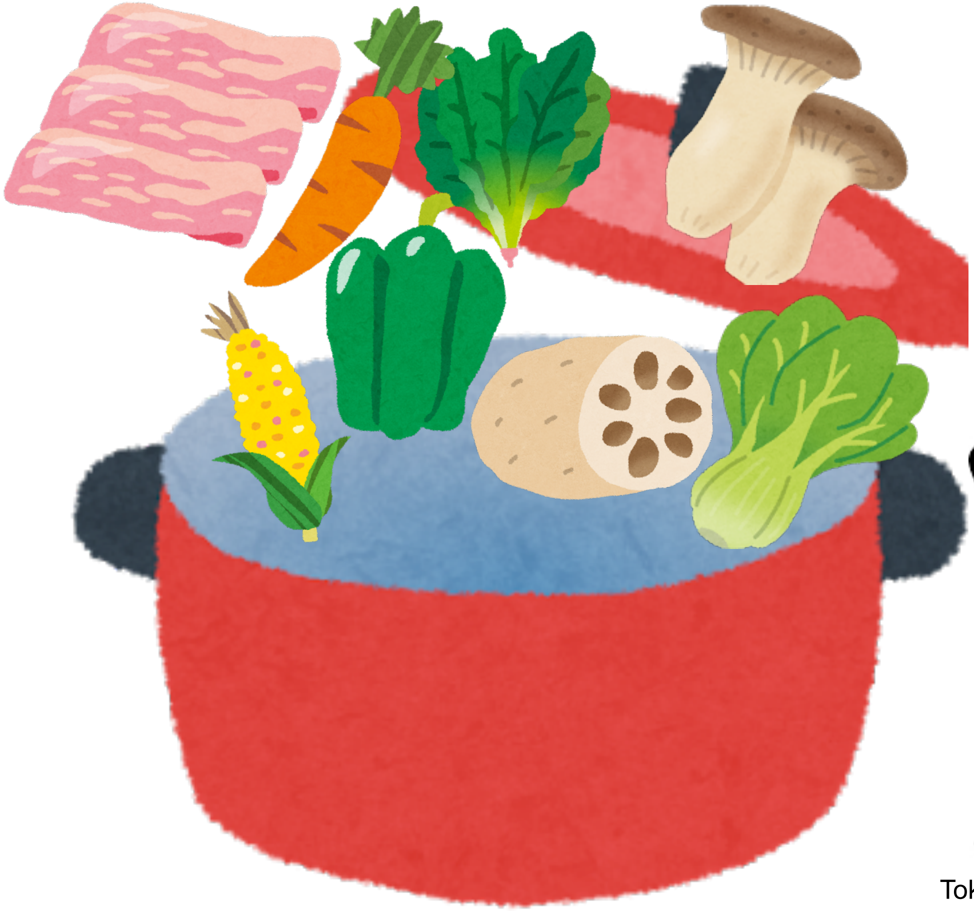

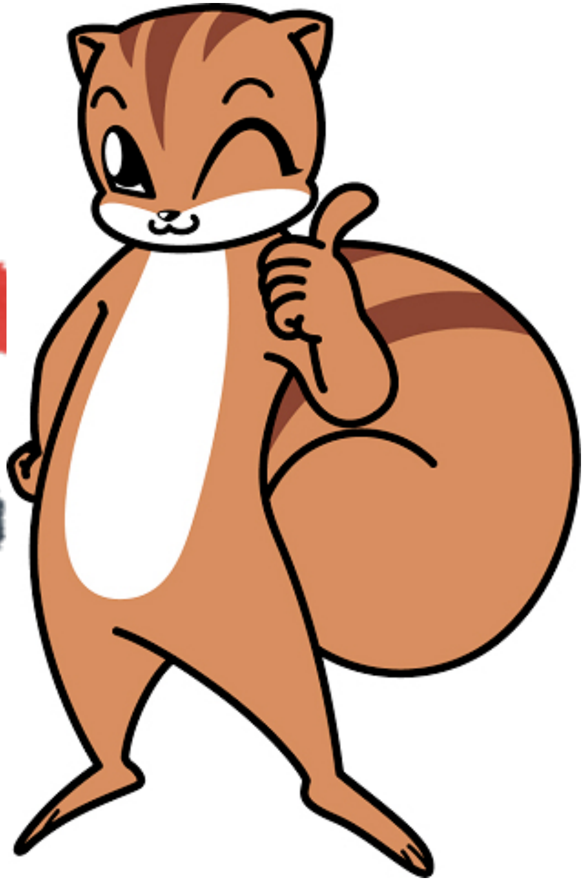

Tokigawa character mascot: Minami-kun

Eating Behavior 4: Promote eating all three meals regularly

When you wake up, perform some light exercise and eat a tasty breakfast.
Eating properly starts with a good breakfast!

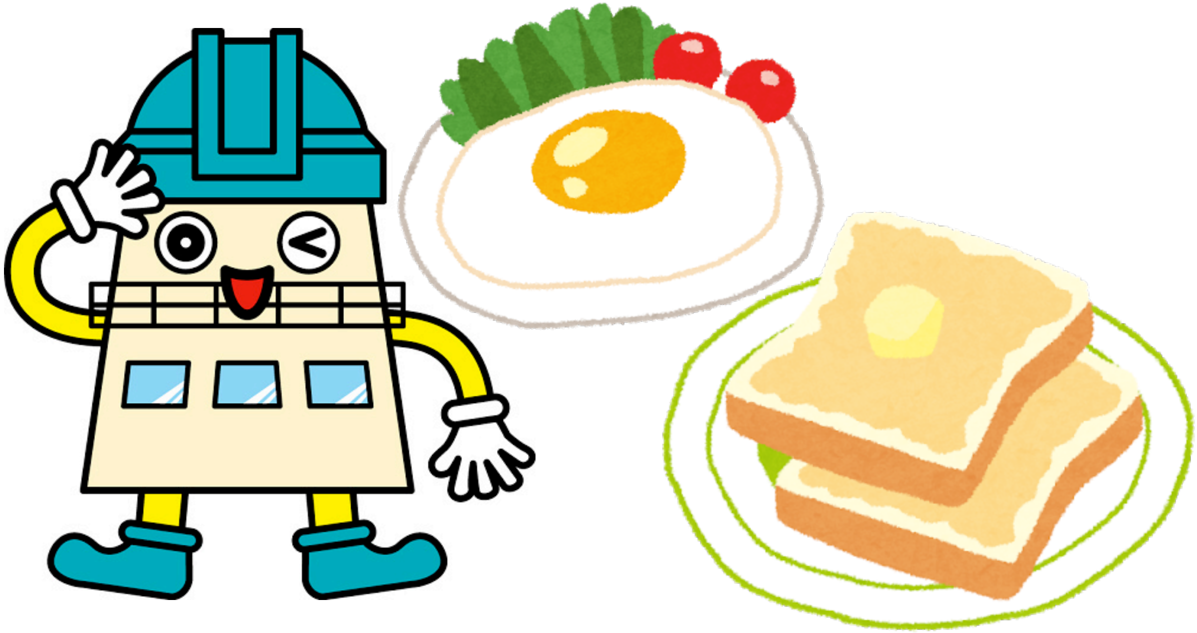

Tokigawa character mascot: Doma-kun

Eating Behavior 5: Promote eating vegetables first

Start your meal with a few fresh vegetables.
That will make it harder for your body to retain fat.

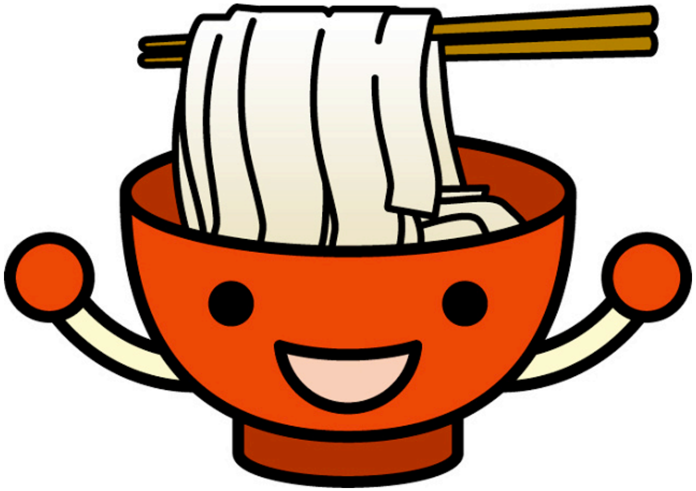

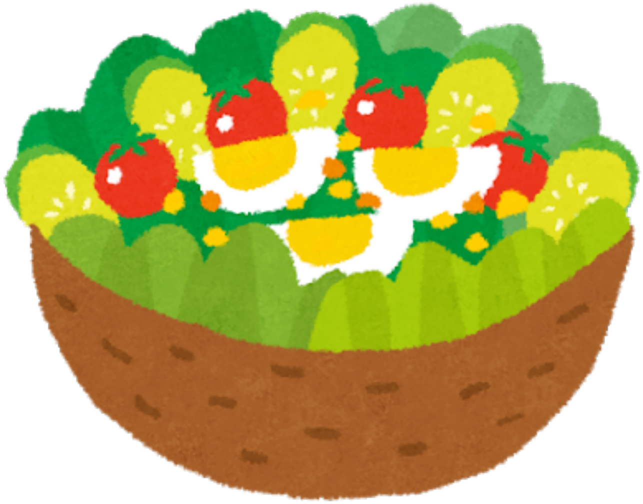

Tokigawa character mascot: Himokawa-san

Eating Behavior 6: Promote eating less

At mealtimes, eat in moderation and take your time.

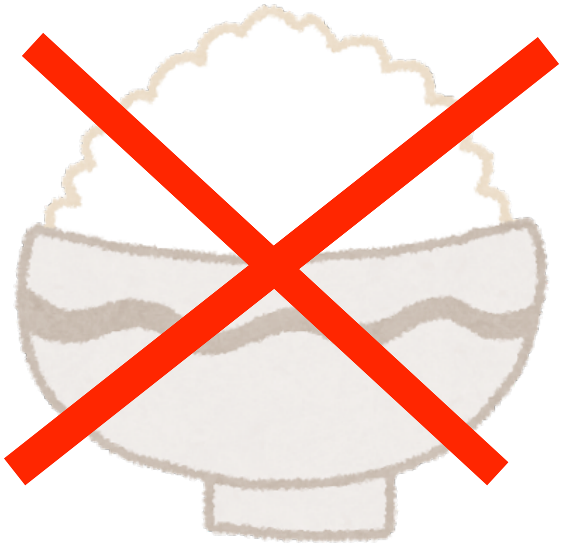

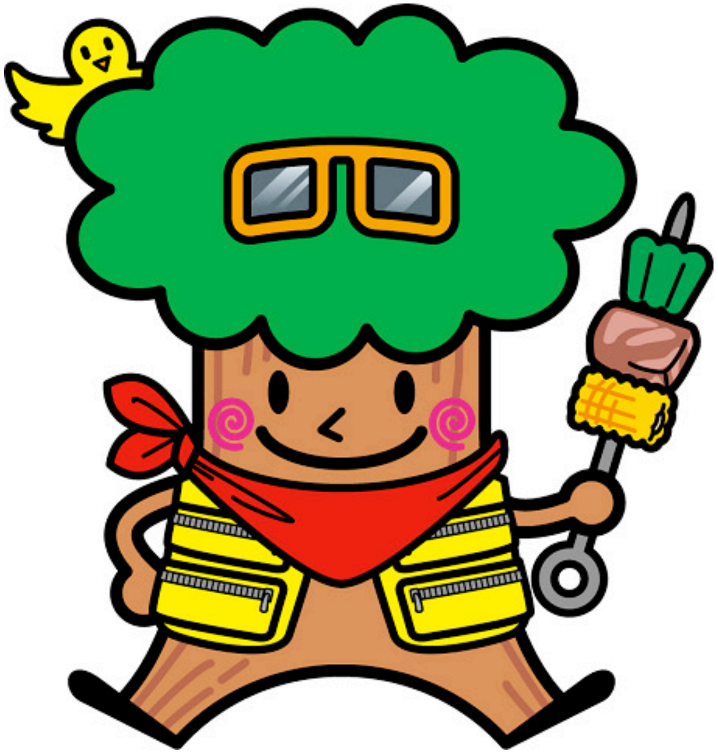

Tokigawa character mascot: Woodman

Eating Behavior 7 : Promote chewing thoroughly

Take your time over your meal. Relax and talk with your friends or family, even if only on weekends.

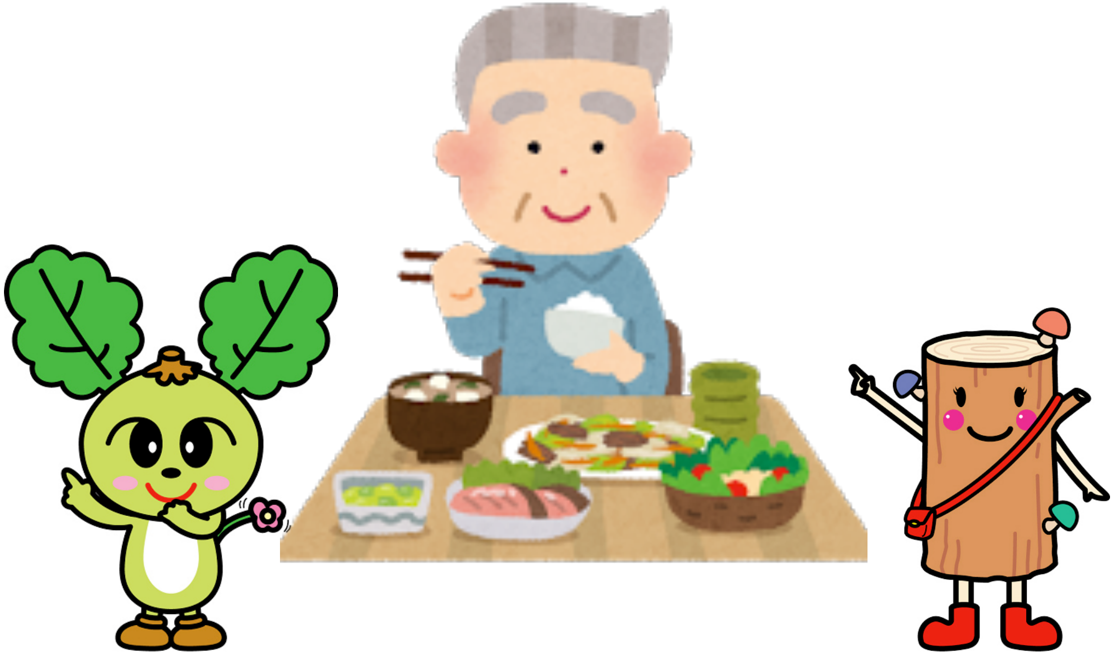

Tokigawa character mascots: Norabitan and Kinoko-chan (Little Mushroom)

Eating Behavior 8: Promote eating salty food in moderation

For seasoning, you can use lemon juice or low-salt miso!
Try it!

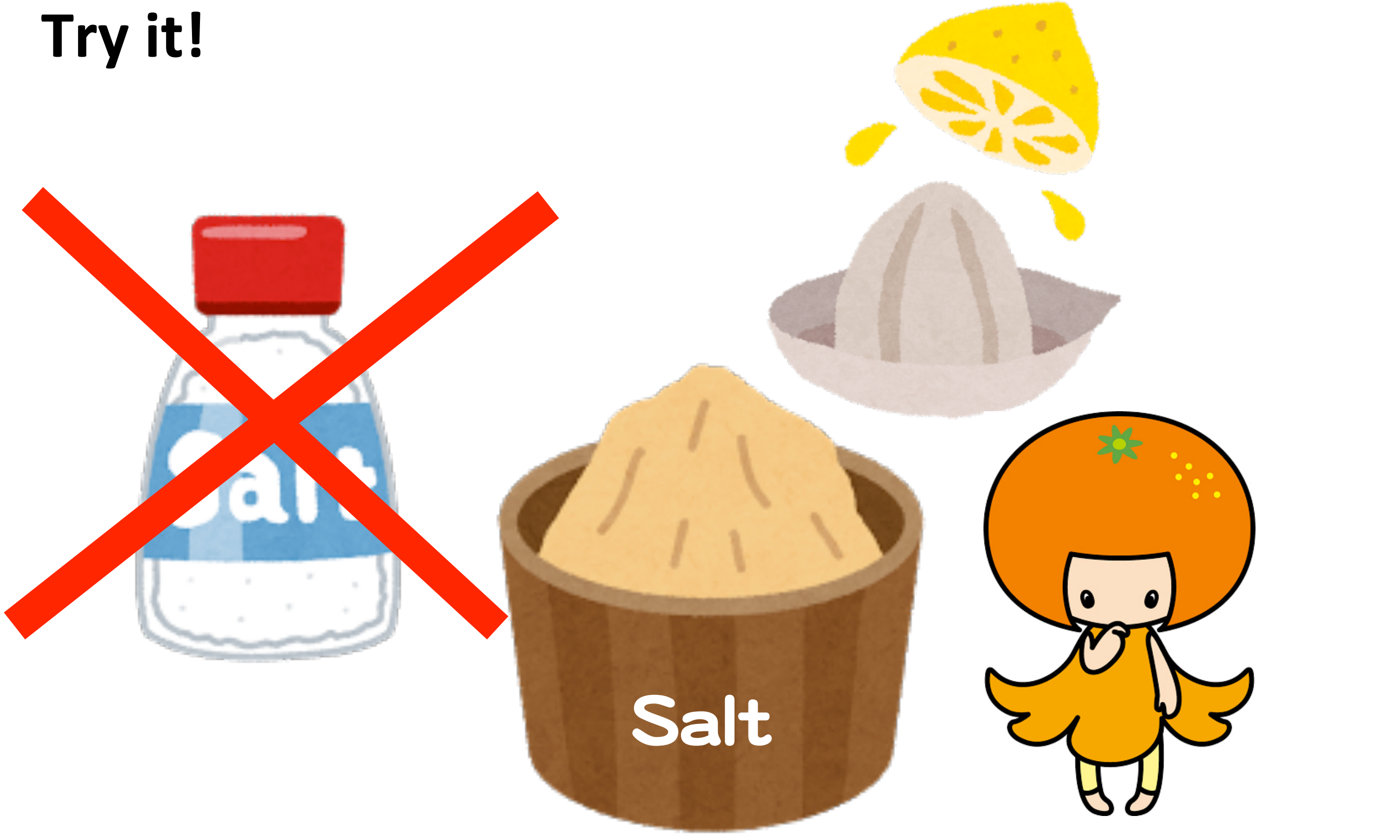

Tokigawa character mascot: Mikan-chan (Little Satsuma)

Eating Behavior 9: Promote eating fatty food in moderation

Try cooking with chicken. It’s good for your body and for the family finances!

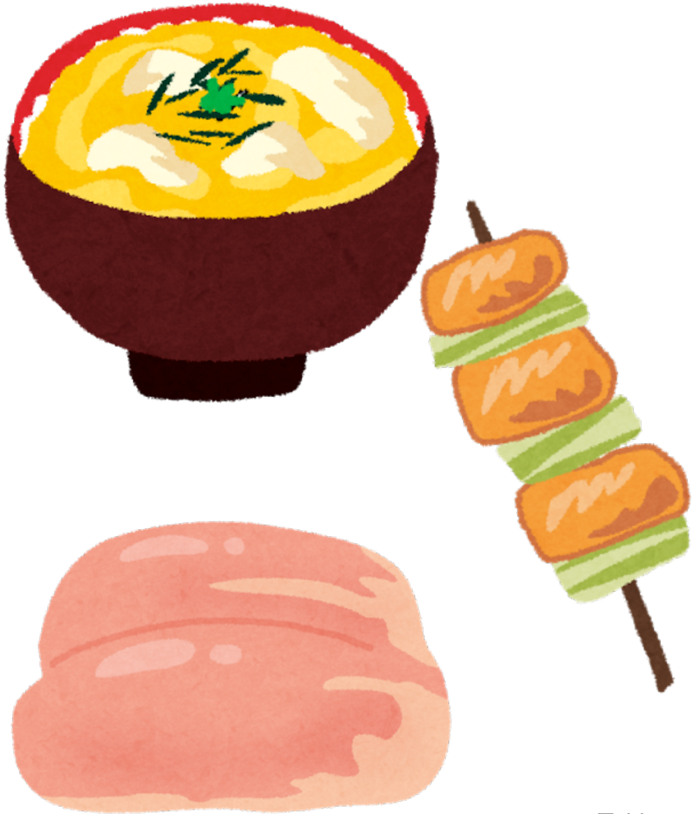

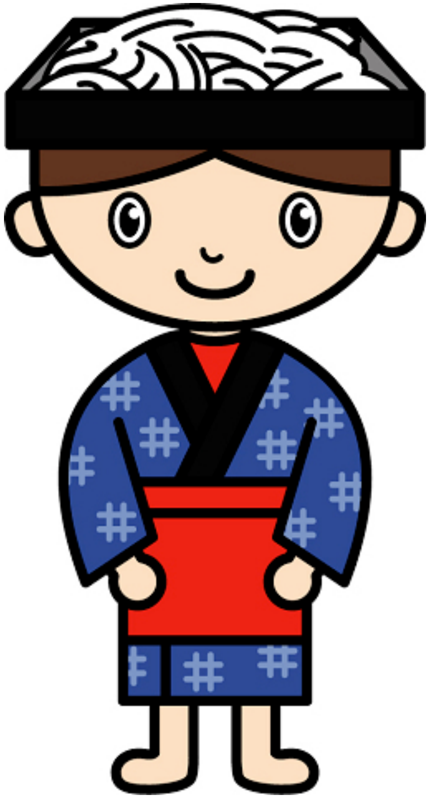

Tokigawa character mascot: Yasuragi-san

Eating Behavior 10: Promote eating sugary food in moderation

Why not try fruit instead of sweets or dessert after your meal?

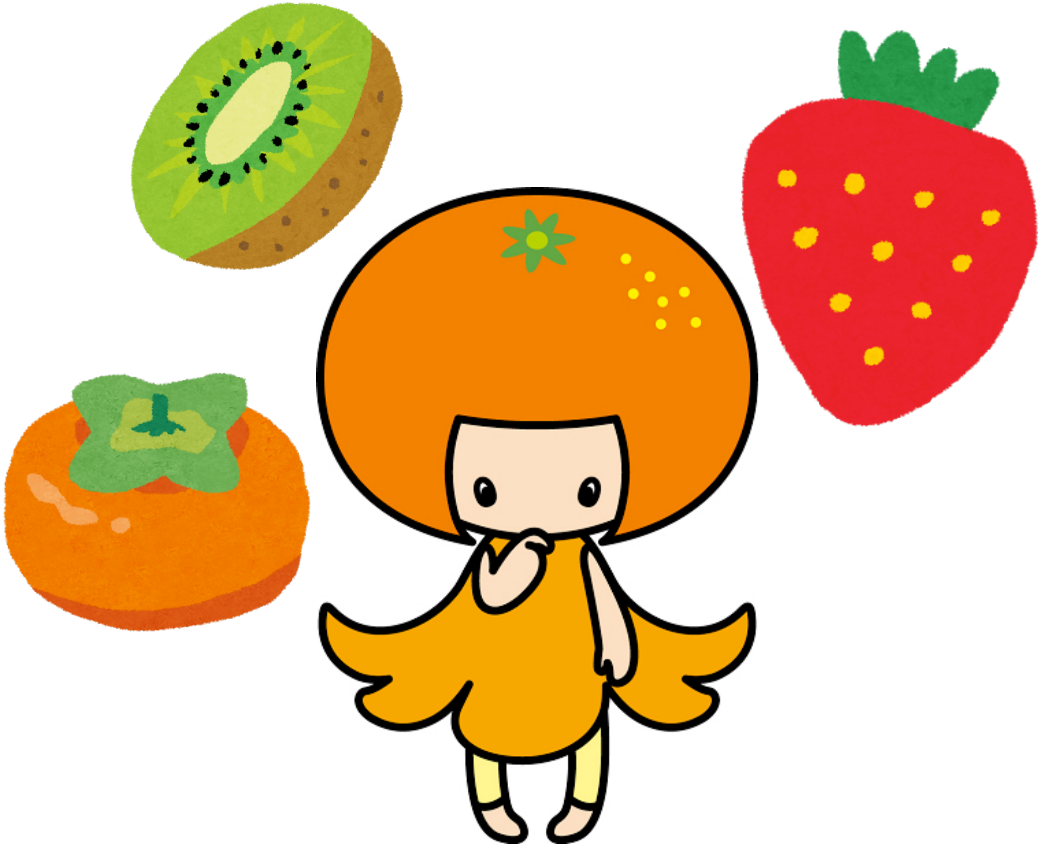

Tokigawa character mascot: Mikan-chan (Little Satsuma)

Eating Behavior 11: Promote avoiding eating between meals

Ask your family to hide sweets and snacks out of sight. When you want to eat something sweet, ask your family nicely to get them out for you!

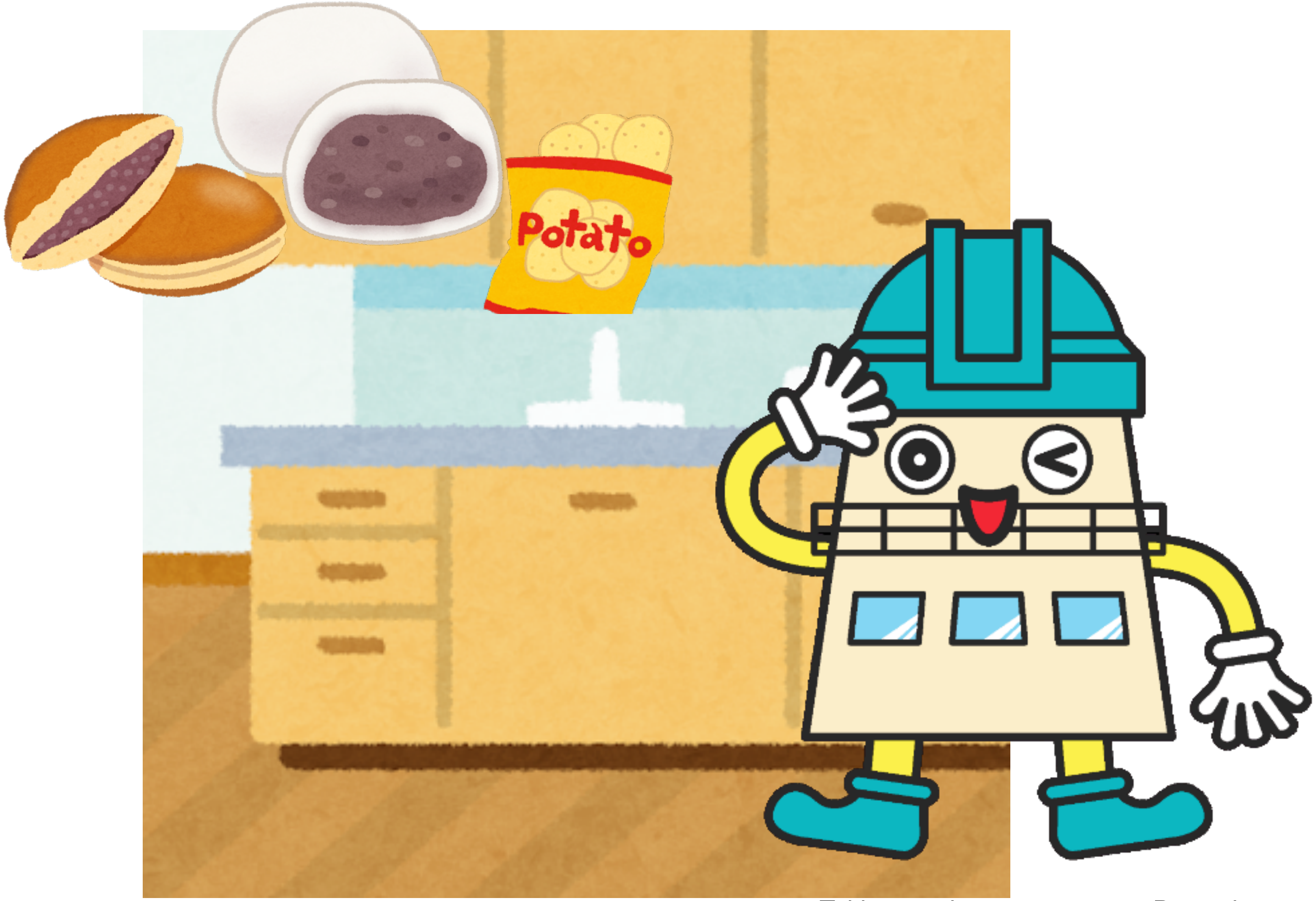

Tokigawa character mascot: Doma-kun

Think about which of these you don't eat very much: carbohydrates, proteins, lipids, vitamins, and minerals

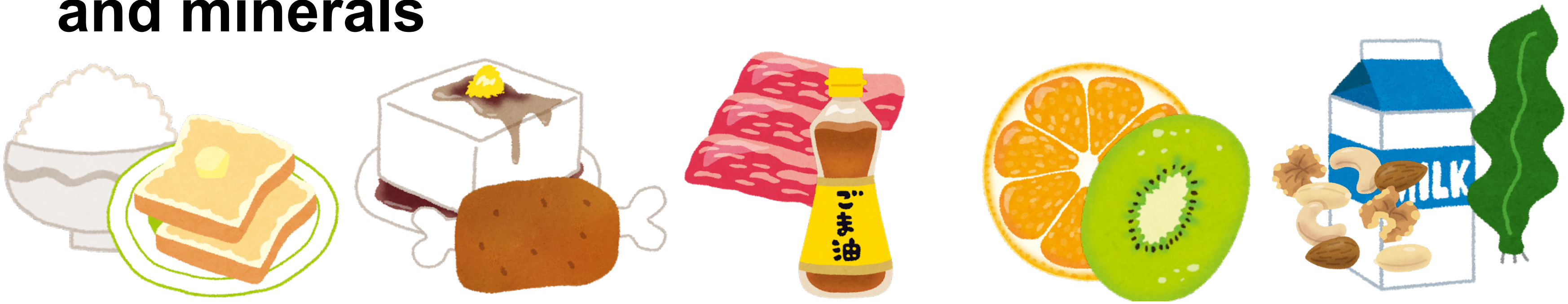

Carbohydrates Protein Lipids Vitamins Minerals

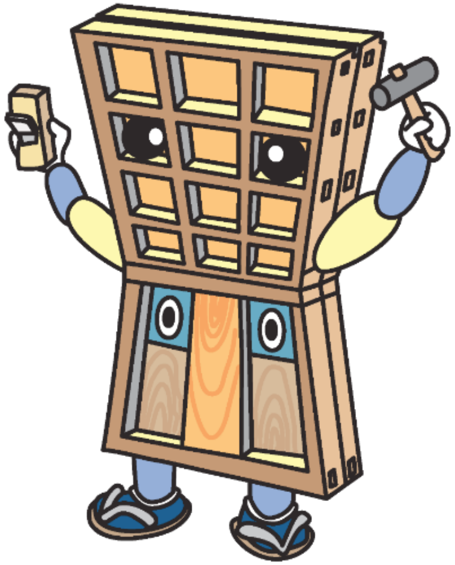

Tokigawa character mascot: Shoji-kun

Japanese Version

フィードバックメッセージ

メッセージは，身体活動，および食行動それぞれの
対象者の実施状況に合わせて選択しました。

【質・量・頻度，いままでよりももう少しだけ変えてみましょう】

暑い夏もしっかり身体を動かしてきた皆さん。秋は体を動かすのに良い季節，今までの積み重ねにさらにもう少しだけスモールチェンジで心も身体も健康に！今の皆さんの毎日を振り返り，下の質・量・頻度を変える方法から，取り入れやすい方法を選んでみましょう。

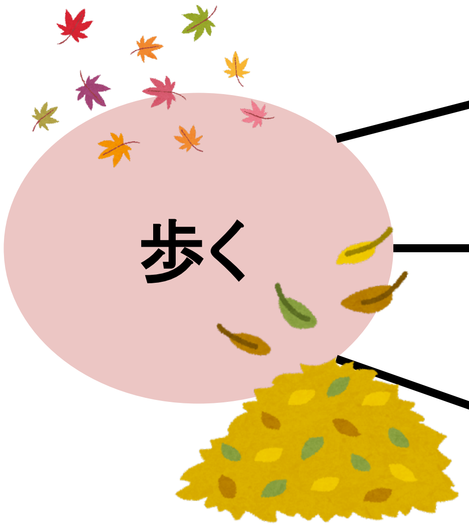

「質（中身）」を変える	早歩きを心がける
「量」を変える	10分を15分に
「頻度」を変える	週1日を週2日へ

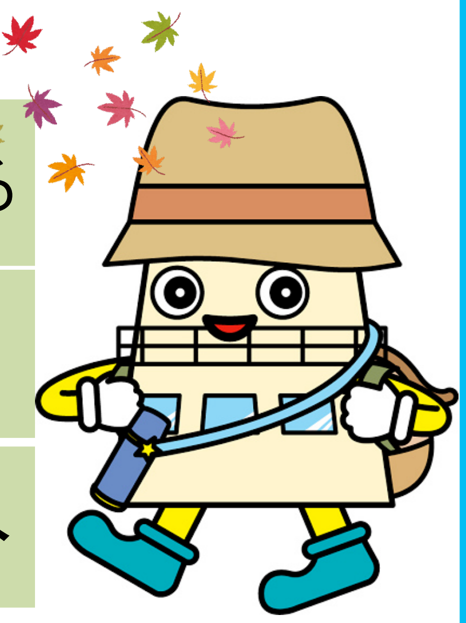

ときがわ町マスコットキャラクター：ドームくん

身体活動1：身体活動量向上者

【天気に負けず！ときがわの秋を楽しみましょう！】

暑い夏をはさみ．せっかく意識して行っていたウォーキングや体操もつらくなり，やめてしまった方も多いはず。これから身体を動かすのにもよい季節になっていきます。友人とのウォーキングや体操，ときがわの自然を楽しみながら心も身体も活動的に。また，雨への対処も忘れずに。家でできる体操や簡単な筋トレを準備しておきましょう。

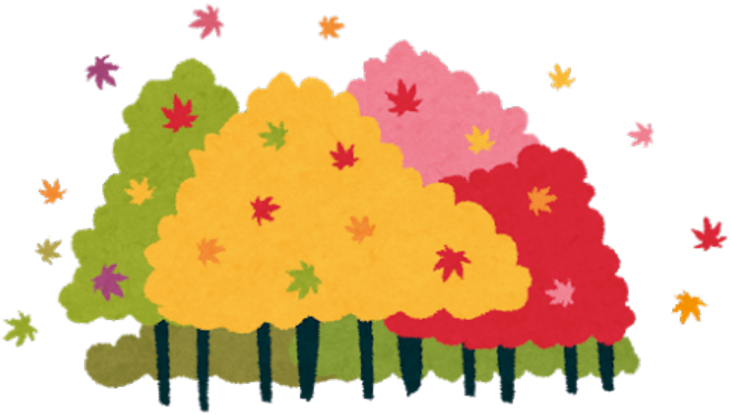

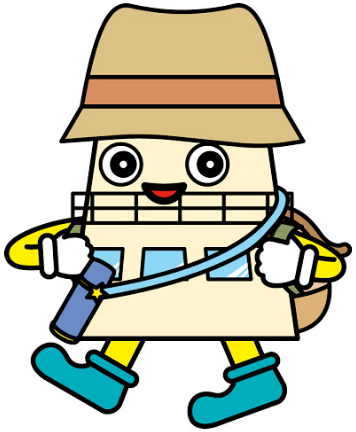

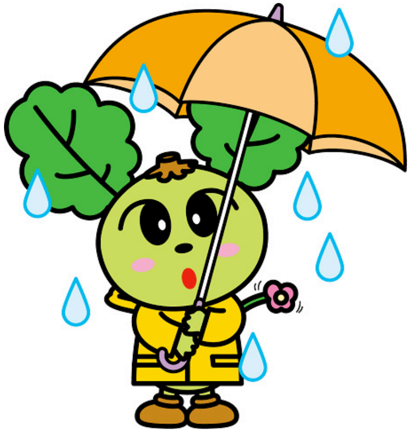

ときがわ町マスコットキャラクター：ドームくんとのラビたん

食行動1：野菜を食べる。

お味噌汁に一工夫！
たくさん野菜を入れましょう。

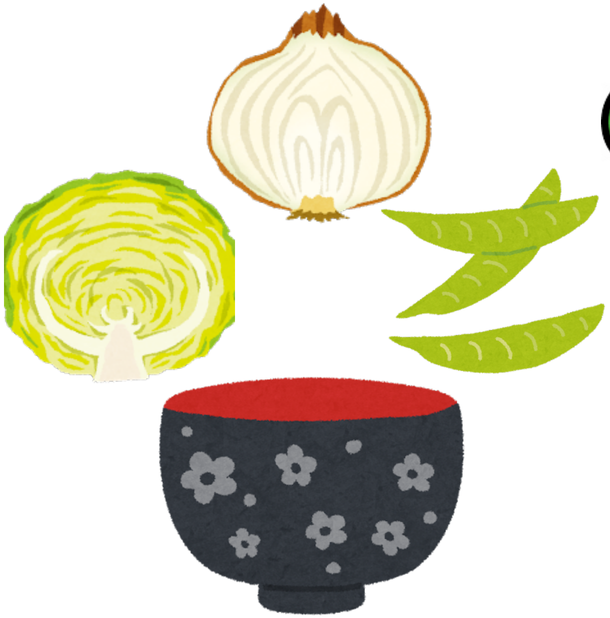

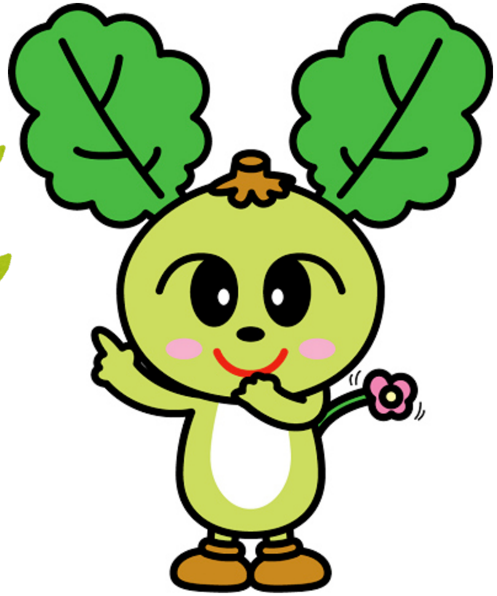

ときがわ町マスコットキャラクター：のラビたん

食行動2：健康に良いと思う食品を食べる。

ヨーグルトや青魚，まずは身体に
良さそうな食品を挙げてみましょう。

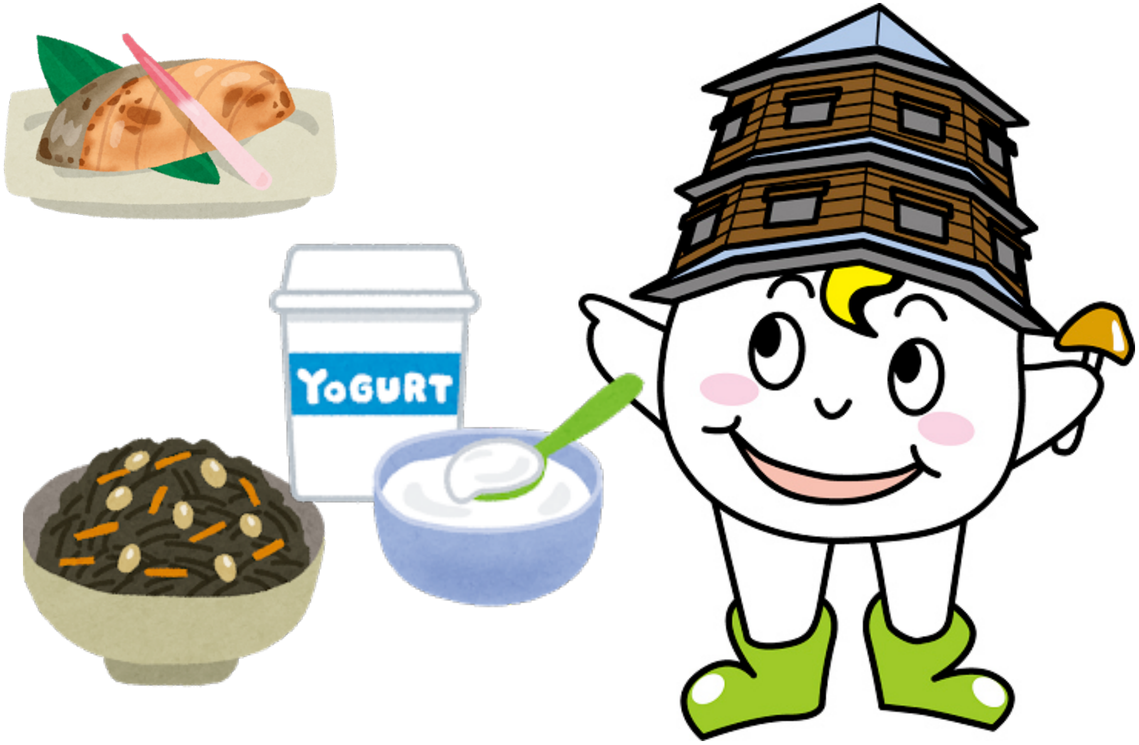

ときがわ町マスコットキャラクター：大野くん

食行動3：多くの品目をとる。

たくさん食品を一気に蒸す！
小食でもあっさり簡単にたくさん食べれます。

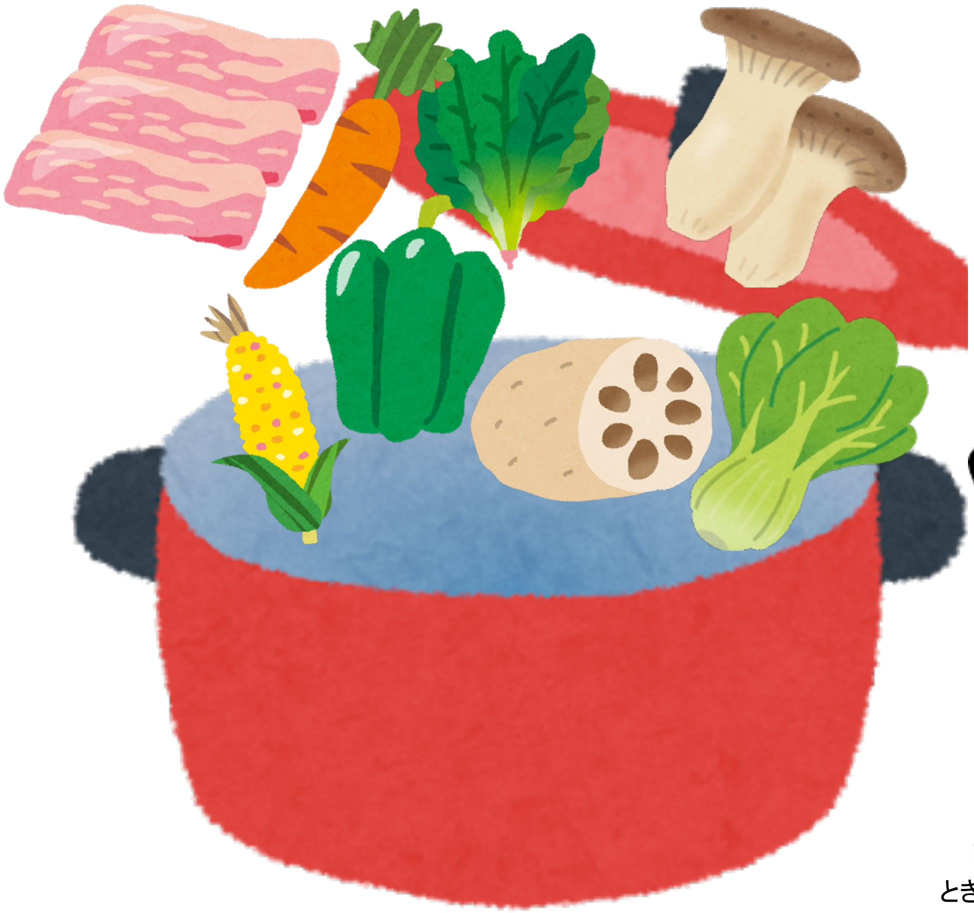

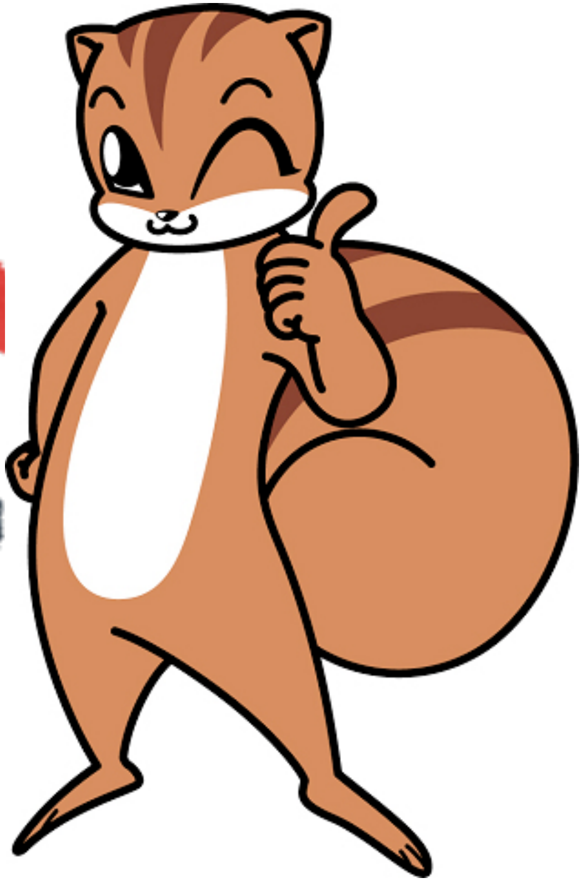

ときがわ町マスコットキャラクター：三波（みなみ）君

食行動4：三食規則正しく食事をしている。

起きたら軽く体操おいしく朝ご飯。
規則正しい食事は朝食から！

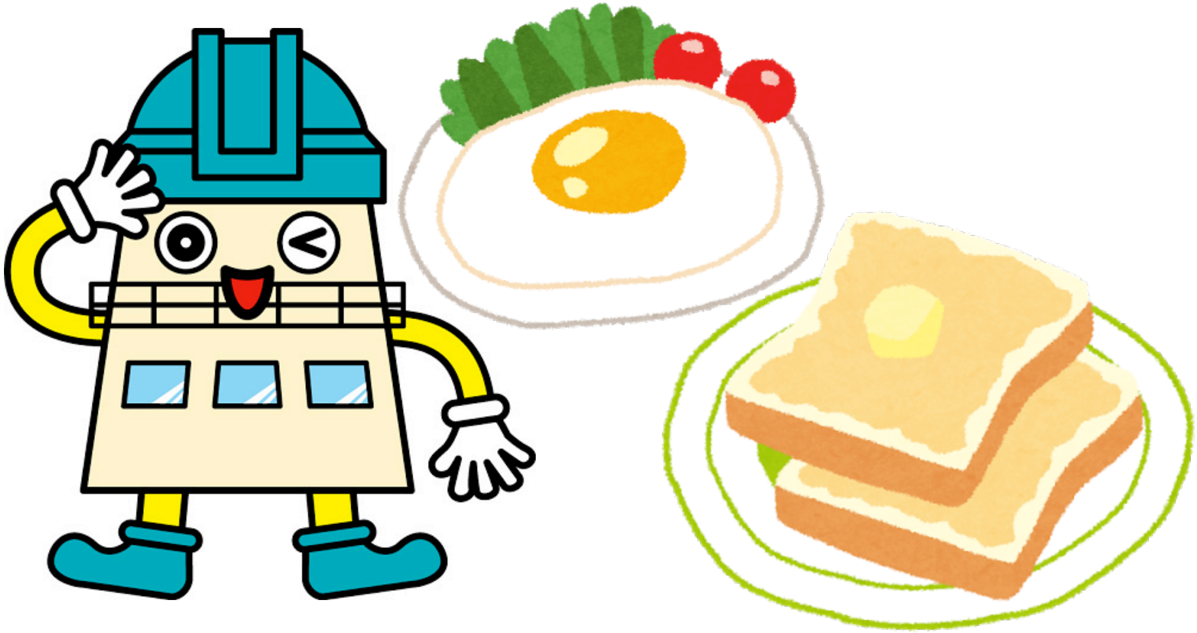

ときがわ町マスコットキャラクター：ドームくん

食行動5：野菜から食べる。

身体に脂肪をつきにくくする。
ご飯のまえにまず少しの野菜から。

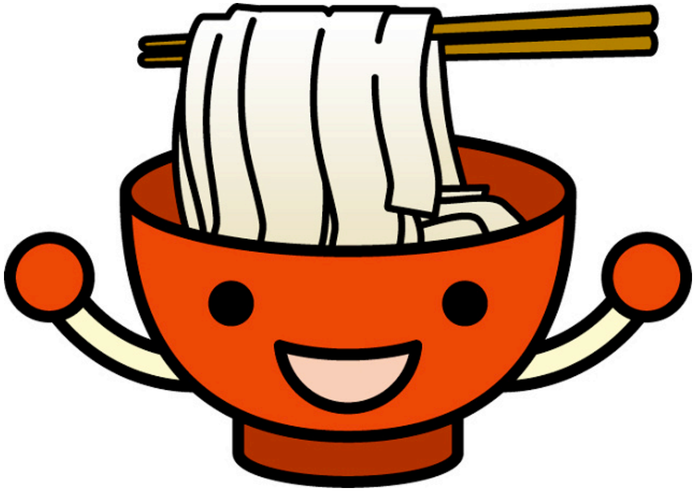

とき がわ町マスコットキャラクター：ひもかわさん

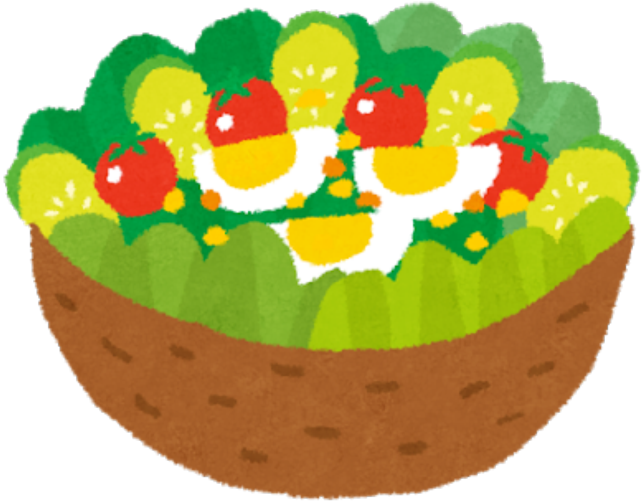

食行動6：食べ過ぎないよう、腹八分目を心がける。

よそるときから八分目，ゆっくり食べましょう。

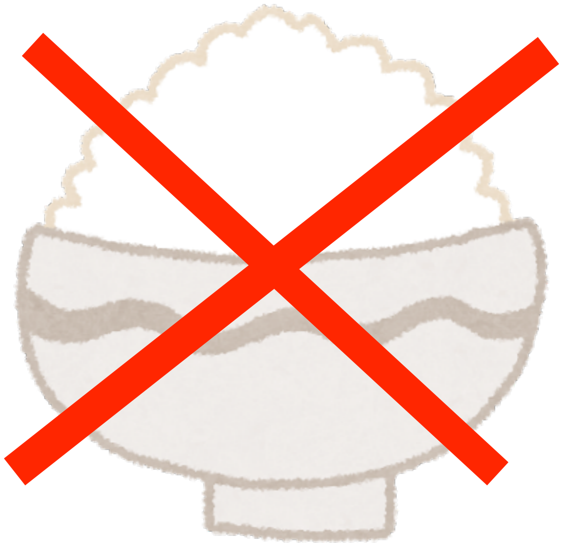

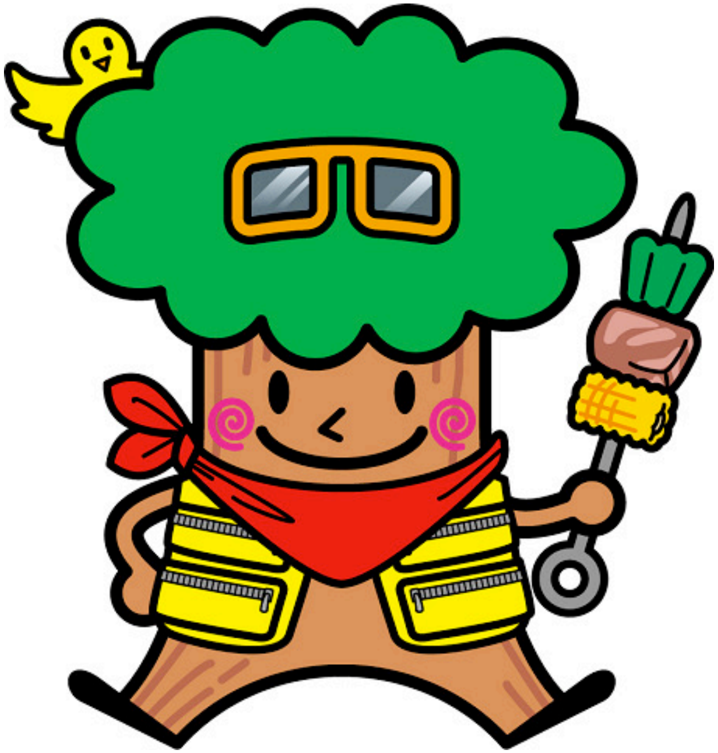

ときがわ町マスコットキャラクター：ウッドマン

食行動7：よく噛んで食べる。

週末だけでも家族や友人とゆっくり
話をしながら，時間をかけて食事をしましょう。

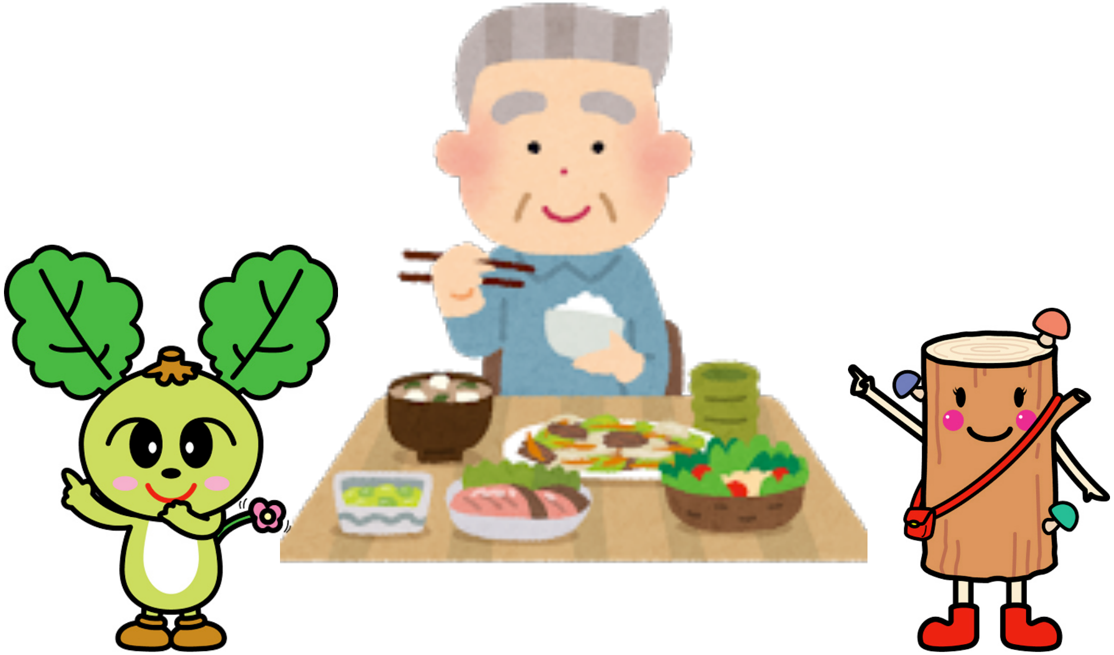

ときがわ町マスコットキャラクター：のラビたんとときのこちゃん

食行動8：塩分を控える。

味付けにはレモン汁，減塩みそ！
積極的に使いましょう。

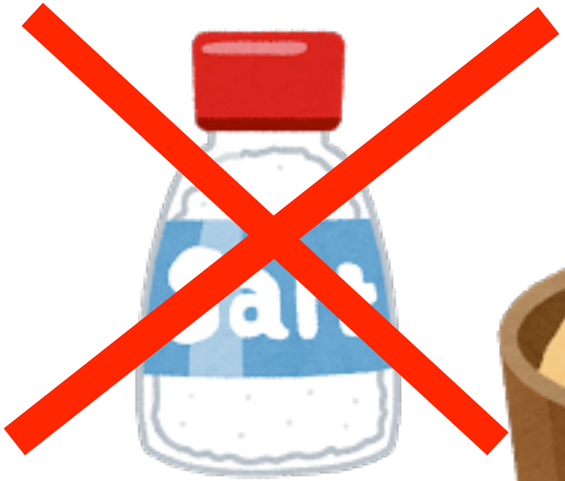

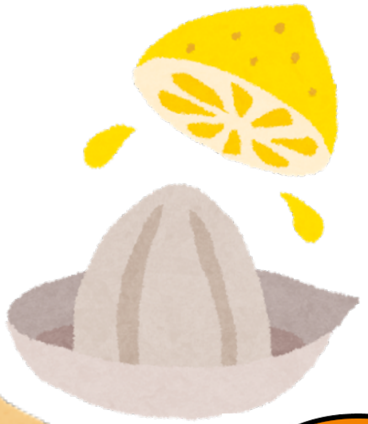

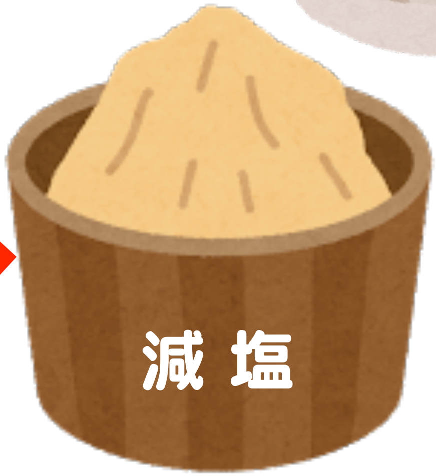

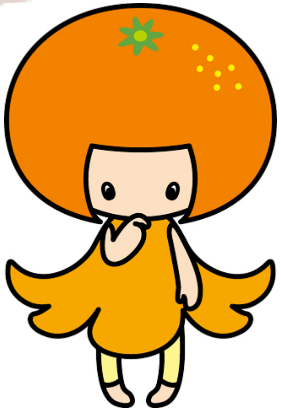

ときがわ町マスコットキャラクター：みかんちゃん

食行動9：油分を控える。

家計にも身体にも優しい！鶏肉料理を！

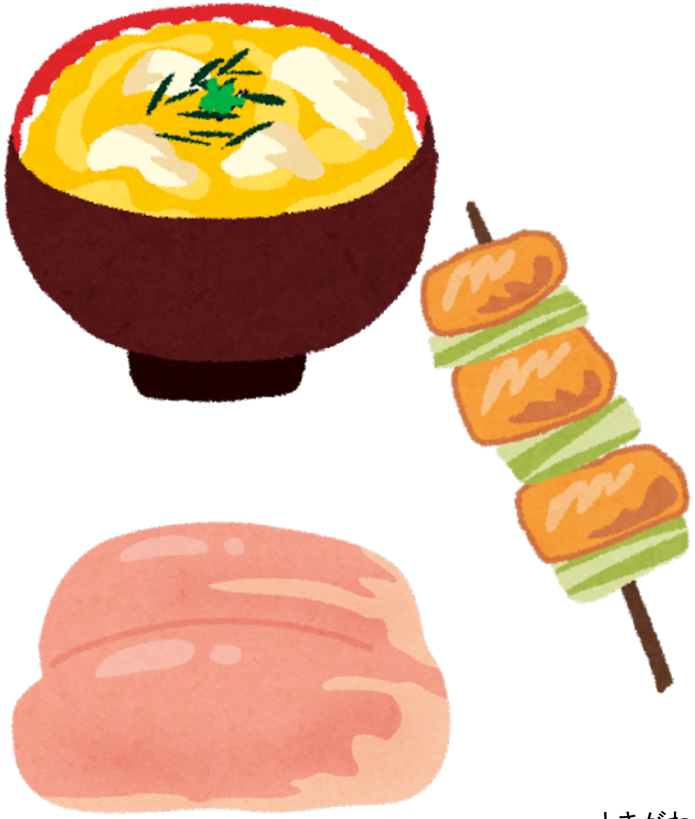

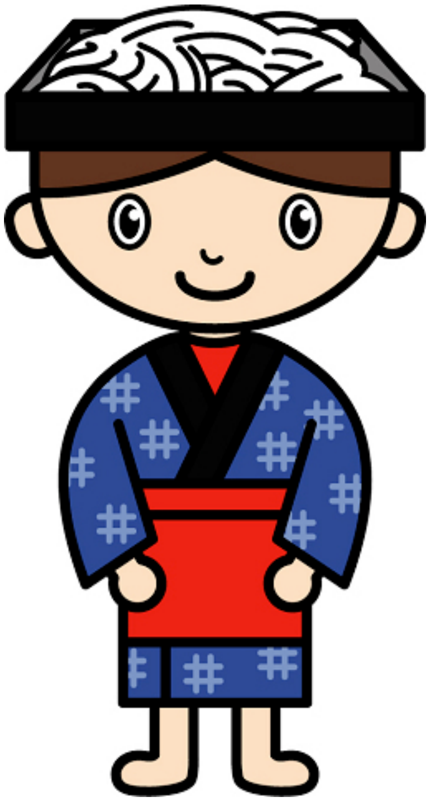

ときがわ町マスコットキャラクター：やすらぎさん

食行動10：砂糖を含む食品を控える。

食後のお菓子，果物に変えてみませんか？

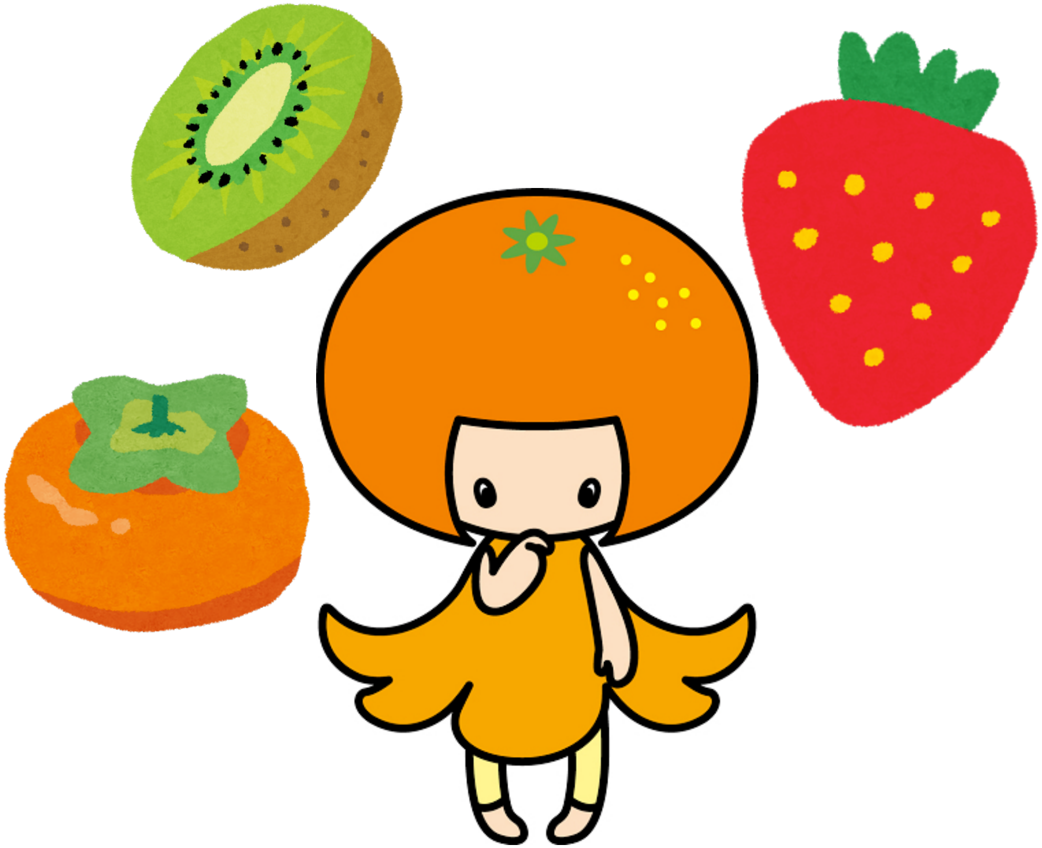

ときがわ町マスコットキャラクター：みかんちゃん

食行動11：間食を控える。

家族に頼んでお菓子を隠してもらいましょう。
食べたいときはお願いして持ってきてもらいましょう！

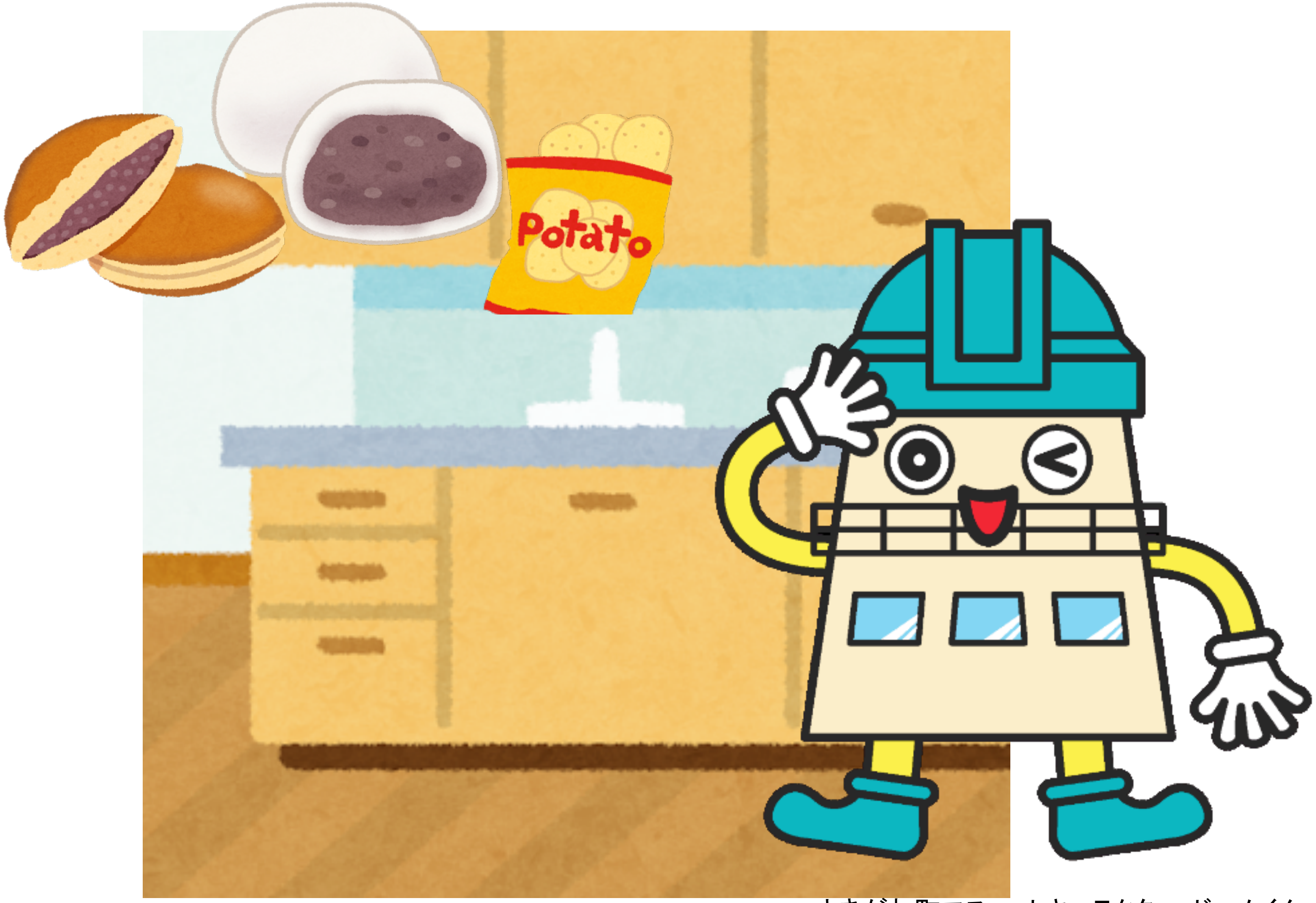

ときがわ町マスコットキャラクター：ドームくん

食行動12:栄養のバランスを考える。

炭水化物，タンパク質，脂質，ビタミン，ミネラル，あまり食べないものはどれか考えてみましょう。

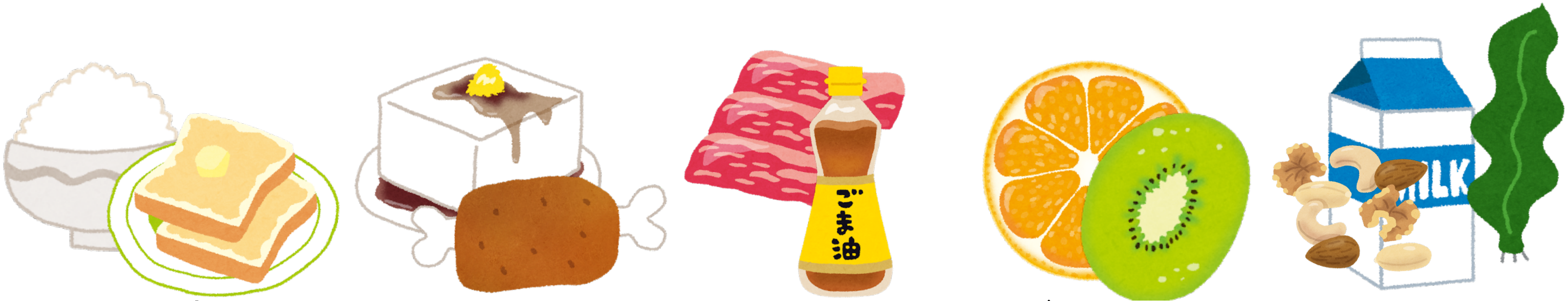

炭水化物　タンパク質　脂質　ビタミン　ミネラル

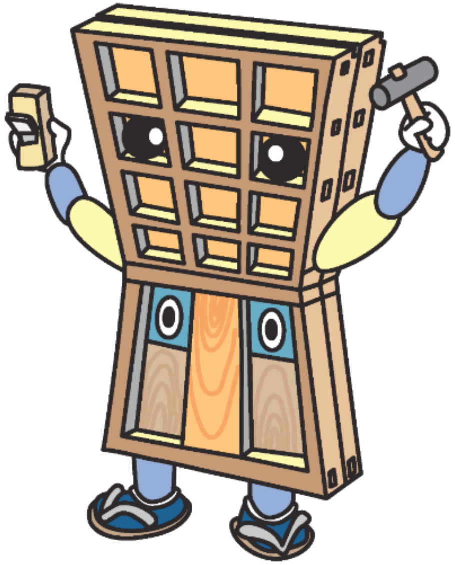

ときがわ町マスコットキャラクター:しょうじくん
